# Mapping Functional Transitions across Protein Homologues from a Single Simulation: Application of gEDES-HM to Adenylate Kinases

**DOI:** 10.1021/acs.jcim.6c02521

**Published:** 2026-10-05

**Authors:** Han Kurt, Mohd Athar, Andrea Basciu, Victor Reys, Alexandre M. J. J. Bonvin, Attilio V. Vargiu

**Affiliations:** † Physics Department, 3111 University of Cagliari, Cittadella Universitaria, Monserrato, Cagliari I-09042, Italy; ‡ WAY4WARD, via Ruggero Bonghi 11/B, Roma 00184, Italy; § Bijvoet Centre for Biomolecular Research, Faculty of Science - Chemistry, 84889 Utrecht University, Padualaan 8, Utrecht 3584 CH, The Netherlands

## Abstract

Proteins fluctuate across different conformational states, which constitute a dynamic ensemble that regulates their interaction networks and functions. Predicting the conformational landscape remains a computational challenge, and even more difficult is capturing the link between state distributions and specific functions within protein homologues. Here, we present gEDES-HM (generalized Ensemble Docking with Enhanced sampling of pocket Shape-Homology Modeling), a scalable computational protocol enabling us to remap conformational ensembles obtained from enhanced sampling simulations of a single protein to its homologous family members, removing the need to perform individual simulations for all of them and thus speeding up the whole process by orders of magnitude. Specifically, we exploit our recent gEDES protocol to bias through metadynamics simulations the shape and volume of the binding site(s) of a reference protein. Next, a number of maximally diverse conformations of the protein are extracted from a multistep cluster analysis and used together as a multitemplate library for comparative modeling of the conformational ensemble of each homologue in the pool. This enables the functional conformational ensemble of one protein to be transferred to its homologues and used, for example, in restrained ensemble docking. We validated the protocol on 22 homologues of the *E. coli* adenylate kinase (hereafter, ADK), corresponding to 26 protein-ligand complexes and spanning 31% to 99% sequence identity to the reference. Our protocol retrieves a bound-like conformation of the protein binding site (corresponding to experimental substrate/inhibitor-bound states) for almost all targets, which allows reproducing native-like binding modes for 92% of the investigated complexes in ensemble docking calculations. Validated so far within a single family, gEDES-HM could provide a practical route to generate binding-competent structural libraries for homologues lacking experimental structures suitable for downstream structure-based modeling.

## Graphical abstract

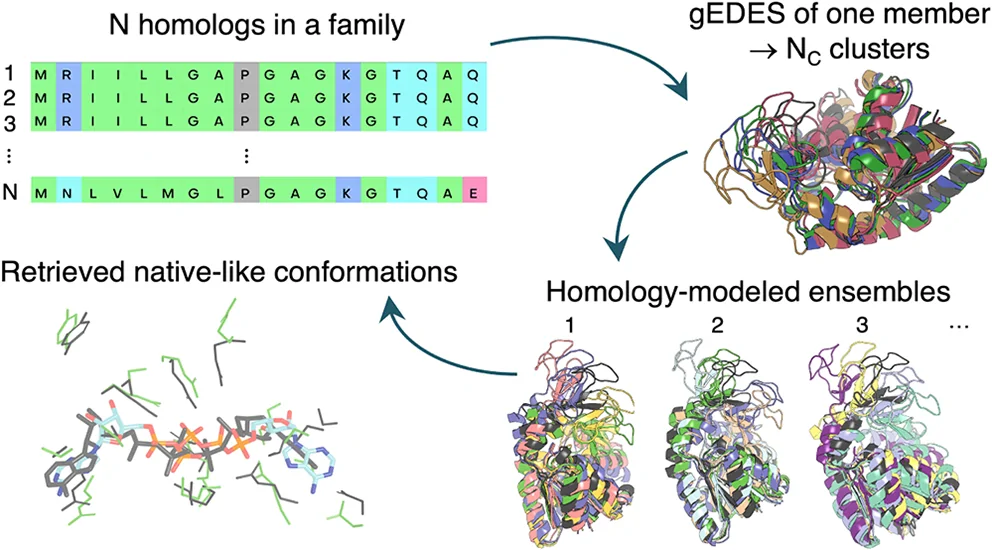

## Introduction

Proteins exist as dynamic ensembles, continuously fluctuating around different conformational states that are essential for their function.^1^ The specific conformational landscape associated with each protein under specific conditions dictates its pattern of interactions, regulating processes such as ligand recognition and allostery.^2,3^ In fact, understanding molecular recognition events that regulate biological processes such as catalysis and cell signaling requires the identification of the most likely metastable states as well as of the transition pathways between them.^4^ Moreover, alterations of the thermodynamics or kinetics relationships among these states can lead to severe pathological conditions.^5,6^

AI-based tools have achieved unprecedented accuracy in predicting the structures of proteins and their complexes,^7,8^ yet most methods primarily yield single structural snapshots or thermodynamic averages, often neglecting conformational states that are underrepresented in the training set but relevant for protein function.^9,10^ Recent analyses confirm that conformationally dynamic proteins frequently evade accurate structural prediction by these tools and that approximately one-third of residues in these models may lack atomistic precision in flexible or modified segments.^11,12^ To move beyond static predictions, a growing class of generative approaches aims to sample conformational ensembles directly rather than return a single structure. Methods such as AlphaFlow extend AlphaFold-like architectures with flow-based objectives to generate sequence-conditioned structural ensembles,^13^ while distributional models such as BioEmu aim to approximate the equilibrium conformational distribution of proteins at scale.^14^ Related simulation-free approaches, including Str2Str, further explore zero-shot conformational sampling from static structural inputs.^15^ Nevertheless, these methods remain constrained by the conformational diversity of their training data, often underrepresenting high-energy regions of conformational space and rare, lineage-specific structural states that deviate from the consensus; furthermore, they generally lack the physical rigor needed to recover quantitatively reliable thermodynamic populations along functionally relevant transitions without additional physical modeling or reweighting.^16^

Prior to the advent of AI-based methods, sampling of biologically relevant conformations has historically been achieved via molecular dynamics (MD) simulations. However, as the simulation time remains a major bottleneck for standard MD,^17^ several enhanced-sampling methods were developed over recent years aiming to improve the description of both the thermodynamics and the kinetics of molecular recognition.^18^ To explicitly account for protein flexibility in molecular recognition, we recently developed gEDES (generalized Ensemble Docking with Enhanced sampling of pocket Shape),^19–21^ a protocol based on metadynamics^22^ to generate druggable-like conformations (to be employed, for instance, in ensemble docking and virtual screening). By biasing pocket shape and volume descriptors, the method enabled specific conformational changes relevant to ligand binding in several targets including allosteric, multidomain proteins.^19–21^ Along these lines, recent work by Vats et al. demonstrates that such approaches can capture cryptic pocket opening and protein-ligand binding events inaccessible to unbiased simulations.^23^

In this work, we extend our methodology to transfer a conformational ensemble obtained through gEDES on a single protein to homologous members of the same family. For this purpose, we apply homology modeling to a set of maximally diverse structures extracted from the gEDES trajectories of the simulated “parent” protein. Homology modeling leverages the principle that structure, and by extension conformational dynamics, is more conserved than sequence.^24^ Traditional homology modeling typically uses a single static template structure, potentially missing the conformational diversity essential for function. Interfacing structure modeling with ensemble docking has shown promise in capturing protein flexibility for drug discovery applications.^25^

The adenylate kinase (ADK) family provides an ideal testing ground for conformational ensemble transfer across homologues using our methodology. ADK enzymes catalyze the reversible reaction 2 ADP ↔ ATP + AMP and share a conserved architecture despite sequence variations.^26–28^ ADK function requires large-scale conformational changes between open and closed states,^19^ whose dynamic transitions have been recently characterized by ensemble-based approaches.^29,30^ The protein is also a metalloenzyme in its working cycle: the physiological phosphoryl transfer requires a Mg^2+^ ion, which brings the donor and acceptor phosphates into reactive proximity, lowers the enthalpic barrier of the reaction, and broadens the transition-state ensemble.^31^ The rationale behind our approach that we name gEDES-HM (where HM stands for Homology Modeling) is simple: rather than running computationally expensive enhanced sampling simulations for every ADK homologue, we propose using the conformational ensemble generated from gEDES simulations of a single representative ADK as a template pool for homology modeling across the entire family. In particular, we hypothesize that conformational clusters resembling ligand-bound (holo) states, as identified by gEDES, can serve as structural templates to generate functionally relevant (druggable) conformations for ADK homologues. This approach establishes a scalable pipeline that leverages the transfer of conformational ensembles across homologues, e.g., to assist structure-based drug discovery. In what follows, we describe and validate gEDES-HM on members of the ADK family. While its transferability to protein families whose functional motions differ from the ones sampled for the reference system (*E. coli* ADK) remains to be established, the protocol achieves very good performance in reproducing functional states across the conformational ensemble of the systems investigated here.

## Methods

### Selection of *E. coli* ADK Homologues

The structures of the protein homologues of *E. coli* ADK were retrieved from the RCSB Protein Data Bank (PDB).^32^ ADK from *E. coli* (PDB IDs of the open and closed conformations 4AKE and 1AKE, respectively) was selected as the reference system (template) because it has been extensively characterized and served as a benchmark system for validating the gEDES protocol.^19^ To compile a dataset of homologous targets related to ADK, we performed an advanced search in the RCSB website for structures sharing >30% sequence identity with *E. coli* ADK. This threshold retains diverse family members while remaining above the “twilight zone” of homology modeling, below which fold recognition can be unreliable.^33^

To ensure dataset quality and validation capability, we retained only ligand-bound experimental structures (ligands are reported in Figure 1) covering at least 90% of the corresponding protein sequence (see Table 1). Note that systems 15–20 are short-form adenylate kinases whose native sequences contain 181 to 195 residues, compared with 214 residues for *E. coli* ADK. In particular, these proteins have a shorter LID insertion, whereby 63–72% of the reference LID residues have no corresponding counterpart. LID-based descriptors are therefore computed over the surviving fractions of the domain for these targets. More limited LID deficits are observed for systems 8, 10, and 13, in which the insertion is natively shorter than in the reference systems (see below). This search yielded 22 structures suitable for homology modeling, spanning sequence identities from 30.8% to 98.6% relative to the reference structure (Table 1). Four structures bear two ligands each, respectively, binding at the LID-core and NMP-core interfaces.

**Figure 1:**
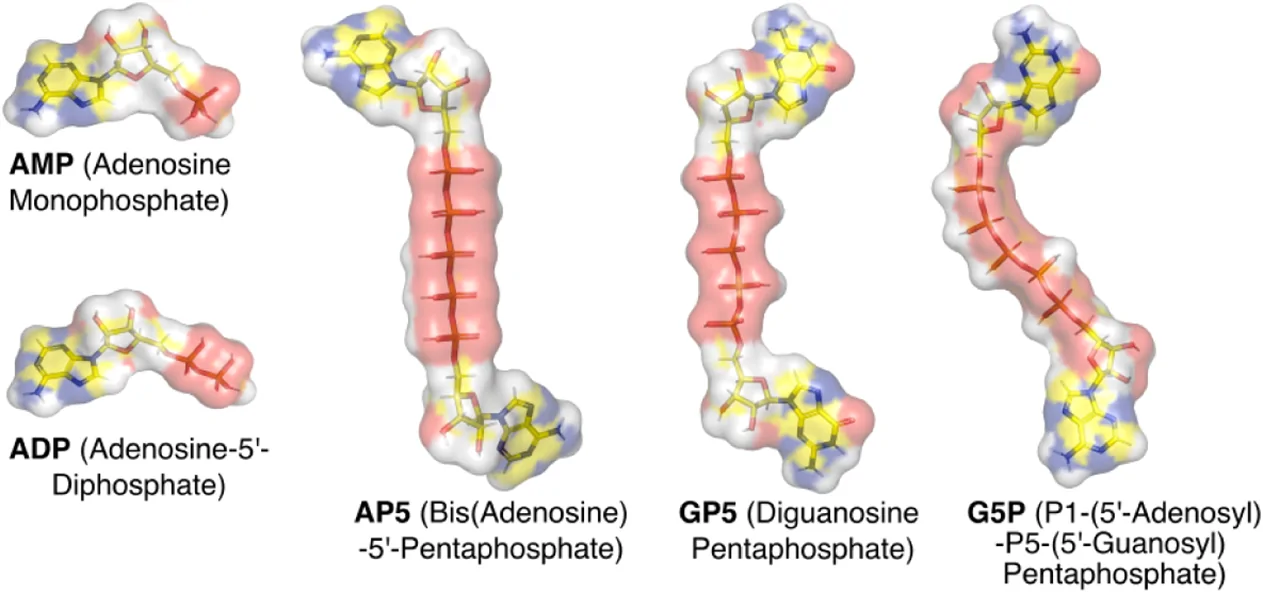
Three-dimensional molecular structures of ligands co-crystallized with the target proteins reported in Table 1. Structures were retrieved from the RCSB Protein Data Bank^32^ and visualized using PyMOL (v3.1).^42^ Carbon, hydrogen, nitrogen, oxygen, and phosphorus atoms are colored yellow, white, blue, red, and orange, respectively. The surface represents the solvent-accessible surface of each ligand using the same color scheme.

**Table 1:** ADK Homologues Selected for This Study^a^

System #	UniProt ID	PDB ID	Ligand	C_α_-RMSD [Å]	Seq length [aa]	% Resolved structure	Seq identity [%]	BS-seq identity [%]
Reference	P69441	1AKE	AP5	-	214	100	-	-
1	A0A0J9X1X4	3X2S	AP5	0.6	214	100	98.6	100
2	Q6LTE1	4K46	ADP and AMP	1.0	214	100	72.9	84
3	P84139	5X6J	AP5	1.4	217	98.2	47.3	84
4	P16304	2ORI	AP5	2.1	217	99.5	46.4	76
5	P07170	1AKY	AP5	1.8	222	98.2	44.5	84
6	P27142	4QBI	AP5	2.1	217	100	44.1	80
7	Q6B341	3FB4	AP5	1.8	216	99.5	47.5	80
8	O66490	2RGX	AP5	2.2	206	98.5	42.5	88
9	Q8IJV6	3TLX	ADP and AMP	2.5	242	97.1	36.4	84
10	P43188	1ZAK	AP5	2.2	222	99.1	39.5	76
11	P08760	2AK3	AMP	7.3	227	99.6	36.8	68
12	Q9UIJ7	6ZJB	G5P	2.6	227	95.6	35.4	68
13	Q04ML5	4NU0	AP5	2.5	212	100	37.4	72
14	P27144	2BBW	GP5	3.8	223	98.7	33.8	64
15	P9WKF5	2CDN	ADP and ADP	2.2	181	100	35.6	64
16	A0A2R2JFU6	5X6L	AP5	2.3	195	95.4	31.3	72
17	Q68EH2	5XZ2	AP5	2.4	194	99.0	31.9	72
18	M4AA20	5YCF	AP5	2.2	193	97.9	32.0	72
19	A0A2R2JFU5	5X6K	AP5	2.3	195	94.9	30.8	72
20	A0A452CSM2	5YCD	AP5	2.3	193	99.0	31.6	72
21	Synth^b^	5G3Y	ADP and ADP	2.2	226	94.2	51.9	80
22	Synth^b^	5G3Z	AP5	2.4	217	99.1	49.1	80

a Structural divergence was estimated from the C_α_-RMSD between the experimental structure of each target (chain A in case of multiple chains) and the reference structure (PDB ID: 1AKE, chain A) to quantify template similarity. The Seq identity [%] displays the percentage identity relative to the reference sequence (P69441), as reported by MView from the full-sequence alignment (see also Figure S1): the number of identical residues divided by the reference length including internal alignment gaps, rather than its ungapped length. The % Resolved structure indicates the fraction of target-sequence residues resolved in chain A of the corresponding structure in the 3rd column, excluding expression tags and cloning artifacts. The BS-Seq identity [%] refers to the percentage of amino acids in the target that are identical to those in the binding site (BS) of the template, the latter being defined as the residues located within 3.5 Å of the AP5 ligand in the 1AKE structure (see Table S1).

b Structures 21 and 22 are synthetic constructs and lack UniProt identifiers.

### Homology Modeling

#### Multiple Sequence Alignment

Target sequences were retrieved from the UniProt database^34^ using the accession numbers listed in Table 1. For synthetic constructs lacking UniProt identifiers (5G3Y and 5G3Z), sequences were extracted directly from PDB files. Multiple sequence alignment (MSA) was performed using the Clustal Omega web server with default parameters, which uses the HMM profile–profile alignment method to achieve high accuracy across distantly related sequences.^35,36^ The resulting alignment was processed with MView^37^ to reformat it and to compute the per-sequence identities relative to the reference reported in Table 1 and was visualized using the ESPript 3.0 web server.^38^ The alignment is provided in Figure S1.

#### Model Generation

The validated sequence alignments were formatted for input into MODELLER (v10.6)^39^ using custom Python scripts. To capture conformational diversity, our modeling strategy leveraged 101 conformational clusters derived from previous simulations of the unbound (apo) *E. coli* ADK (PDB ID: 4AKE).^19^ Note that those gEDES simulations were performed on the apo enzyme in the absence of Mg^2+^, as in previous works by others.^40,41^ Consequently, also the docking calculations were performed in the absence of Mg^2+^; the implications of this choice for the transferred ensemble are discussed in the “Analysis of Unsuccessful Cases and Limitations of Our Approach” section. The 101 clusters span enzyme conformations including open (inactive), partially closed, and ligand-bound-like (holo-like) structures that are typically inaccessible via standard MD simulations.

Prior to model building, terminal regions of the target sequence lacking template support were trimmed from the alignment; only N- and C-terminal regions were removed, leaving all internal gaps intact to avoid introducing unsupported deletions. For homology model generation, we used the “*AutoModel*” class with the Variable Target Function Method (VTFM), which progressively optimizes a spatial restraint-based objective function starting from local interactions and gradually incorporates longer-range restraints.

Geometry optimization consisted of conjugate-gradient minimization followed by molecular dynamics with simulated annealing (*md_level* = *refine.slow*) to ensure thorough conformational refinement. For each of the 101 templates, 10 independent models were generated with different random seeds, yielding an ensemble of 1010 structures per target. This approach resulted in a total dataset of 22220 homology models across the 22 ADK family members, combining diverse template conformations with multiple independent modeling runs. The same workflow was applied to the single-template experimental apo and holo structures (PDB IDs: 4AKE and 1AKE, respectively).

Model quality was assessed using the Discrete Optimized Protein Energy (DOPE) score, a statistical potential that estimates how closely atom–atom distance patterns resemble those observed in native protein conformations, with lower (more negative) scores indicating models with more plausible protein-like geometry.^43^ From the initial pool of 1010 models per target, we selected the best-scoring model (lowest DOPE score) for each of the 101 conformations. This strategy resulted in a final ensemble of 2222 structures (101 models × 22 targets), ensuring that conformational diversity was preserved for subsequent ensemble docking calculations.

Two single-template control sets were built with an identical protocol and the identical alignments. In the first, the experimental apo structure of *E. coli* ADK (PDB ID: 4AKE) was the only template; in the second, the experimental holo structure (PDB ID: 1AKE) was used instead. Ten models per target were retained for each control. Throughout this work, the three model sets are referred to as HM_gEDES_ (101 models per target, selected by DOPE score from the gEDES pool) and HM_4AKE(apo)_ and HM_1AKE(holo)_ (10 models for each target). The HM_1AKE(holo)_ represents the more stringent control, as it incorporates experimental information about the bound state of the reference protein that would not be available in a prospective application and it therefore bounds what the transferred ensemble can be expected to achieve at the modeling stage.

To quantify the accuracy in reproducing experimental structures of the complexes between the targets and their ligands, we evaluated for each model the RMSD values of both the protein (on the C_α_ atoms) and the binding site (RMSD_BS_; heavy atoms) from the corresponding experimental structure of the complex, using in-house *Tcl* (in VMD^44^) and *cpptraj* (in AMBER^45^) scripts. Binding-site residues were defined as those within 3.5 Å of the bound ligand in the corresponding experimental holo structures reported in Table 1.

### Projection of Conformational Ensembles onto Collective Coordinates Describing Open-to-Close ADK Transition

The ADK conformational transition can be described using several collective coordinates.^46–51^ Here, we used two sets of coordinates employed in several earlier works:

a) The center-of-mass distances between the LID and CORE and between the NMP-binding and CORE domains^46–49^ (hereafter *d*_1_ and *d*_2_, respectively; see Figures 2A and S2). To define these coordinates, we adopted the residue ranges used for *E. coli* ADK,^50^ namely, residues 118–160, 30–67, and 1–29/68–117/161–214 for the LID, NMP, and CORE domains, respectively, and computed the two distances using *cpptraj*. The same definitions were mapped onto each homologue through the MODELLER alignment used to build its models. Six targets (systems 15–20) lack between 63% and 72% of the LID residues, so *d*_1_ is computed over the surviving fraction of the domain and is not comparable with the values of the long-form targets; these six targets are nonetheless plotted in Figure 2 for completeness and are labeled accordingly. Additionally, for systems 8, 10, and 13, the LID-involving distances are computed, respectively, over 84%, 91%, and 88% of the LID residues, the remainder having no counterpart in their natively shorter LID insertion.

**Figure 2:**
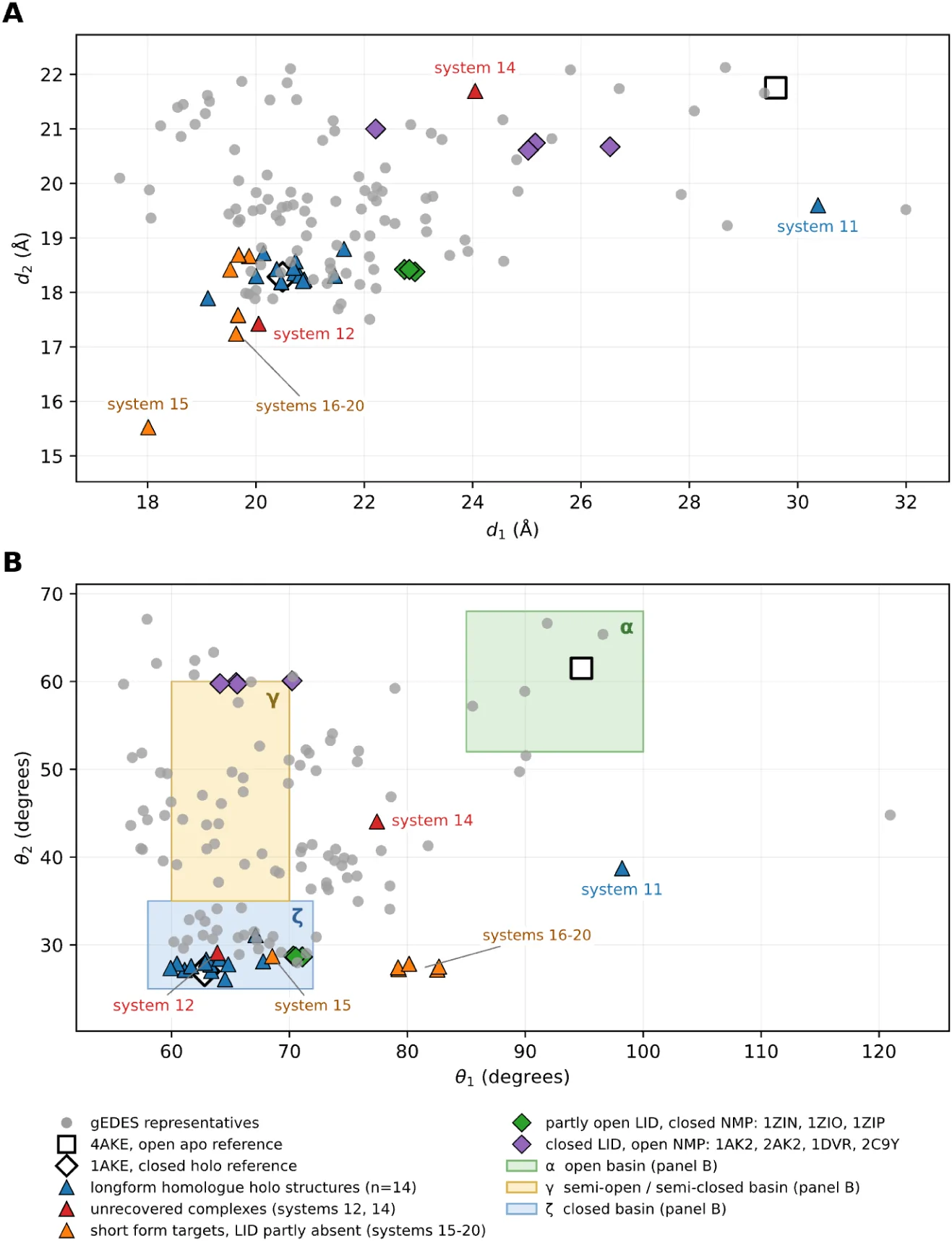
The transferred pool on the two established collective-coordinate descriptions of the adenylate kinase transition. (A) Center-of-mass distance *d*_1_ between the LID and the CORE domains and *d*_2_ between the NMP-binding and the CORE domains, with the domain definitions of Table S3. (B) The LID-CORE angle θ_1_ and the NMP-CORE angle θ_2_ used by Li et al.,^51^ with the three conformational basins they report shaded: α open, γ semiopen–semiclosed and ζ closed. Both panels show the 101 gEDES representatives that constitute the transferable pool, the two *E. coli* references (4AKE open apo and 1AKE closed holo), the holo experimental structures including the two unrecovered complexes, and seven experimental structures that occupy intermediate positions along the transition: 1ZIN, 1ZIO, and 1ZIP, which have a partly open LID with the NMP domain closed,^53^ and 1AK2, 2AK2, 1DVR, and 2C9Y, which have the LID closed with the NMP domain open.^51^ The six short-form targets (systems 15–20) lack most of the LID, so their *d*_1_ and θ_1_ are computed over the fraction of the domain that is present, 28–37% and 6–18% of the residues entering each coordinate, respectively; these two abscissae are therefore not comparable with the other markers, whereas *d*_2_ and θ_2_ are computed over complete domains in every case.

b) We also projected the same structures onto the pair of LID-CORE and NMP-CORE domain angles, which were used by Beckstein et al.^52^ and by Li et al.^51^ to describe the ADK open-to-close transition; here, we adopted the residue definitions from the latter publication. The angle θ_1_ is defined as the one formed by the centers of mass of the backbone of residues 123–155 (LID), 161–165 (hinge), and 1–8, 79–85, 104–110, and 190–198 (CORE). The angle θ_2_ is formed by the centers of mass of the backbone of residues 50–59 (NMP), CORE, and hinge (see Figures 2B and S3). For systems 8, 10, and 13, the LID-involving angles are computed respectively over 79, 88, and 85% of the LID residues. Alongside the reference structures 1AKE and 4AKE, we projected seven additional experimental structures representing putative intermediate conformations along the transition: the *Bacillus stearothermophilus* complexes with Ap5A, Mg-Ap5A, and Mn-Ap5A (PDB IDs: 1ZIN, 1ZIO, 1ZIP), which have a partly open LID with the NMP domain closed,^53^ and the structures corresponding to PDB IDs 1AK2, 2AK2, 1DVR, and 2C9Y, assigned by Li et al.^51^ to the semiopen–semiclosed basin. Homologue and intermediate structures were placed in *E. coli* numbering by global sequence alignment.

### Ensemble Docking

#### Docking Protocol

Ensemble docking was performed using the HADDOCK3 software (v2026.3.0).^54^ The previous version of this software was already successfully employed by us in previous applications of gEDES^19–21^ coupled to protein-small-molecule docking calculations.^55,56^ Ligand topology and parameter files in CNS format,^57^ as required by HADDOCK3, were generated from the crystallographic ligand coordinates using a local installation of PRODRG. Before submission, atom ordering was canonicalized through RDKit^58^ to ensure consistent atom naming between the topology and the coordinate file. The resulting .*top* and .*param* files, together with the ligand PDB containing the standard PRODRG atom names and the original crystallographic coordinates (bound form), were used as the ligand input for all HADDOCK3 calculations. The PRODRG-derived ligand topologies use a united-atom representation in which nonpolar hydrogens are incorporated into the corresponding carbon atom types. Accordingly, the ligand coordinate files contain only heavy atoms with hydrogen atoms subsequently added by HADDOCK3 during topology generation. The same ligand topology was used consistently for each occurrence of a given ligand across all complexes, with 4 polar hydrogens and a net charge of −2 for AMP, 4 polar hydrogens and −3 for ADP, 8 polar hydrogens and −5 for AP5, 9 polar hydrogens and −5 for G5P, and 10 polar hydrogens and −5 for GP5. In ensemble cross-docking, the 101 homology models of the receptor (selected by the DOPE score) were concatenated into a single multi-MODEL ensemble and supplied as molecule 1, with the ligand supplied as molecule 2. As in previous works,^19,20^ the ligand was kept in its reference (crystallographic) conformation during docking, so as to assess whether scalable homology models can by themselves recover the native binding mode using the receptor conformation as the only variable under investigation. Thus, the calculations reported in this work are restrained, receptor-conditioned (not blind) docking runs. This choice is further motivated by two practical considerations: (i) the long polyphosphate bridge of these bisubstrate ligands has torsional and electrostatic parameters that are not yet reliably described by current force fields, so sampling its torsions would inject force-field uncertainty into the RMSD-based metric used to assess model quality; (ii) most ADK family members provide only one or two holo structures (Table 1), providing no experimental reference against which alternative ligand conformations could be validated. Flexible-ligand docking remains better suited to other objectives, such as enrichment-based virtual screening, than to the RMSD-based assessment of receptor model quality pursued here. Docking was driven by ambiguous interaction restraints derived from the experimental binding-site residues, applied as center-of-mass restraints (options *cmrest* and *cmtight*).

The protocol consisted of rigid-body docking, score-based selection, flexible refinement (flexref), and energy-minimization refinement (emref). Rigid-body sampling was set to generate 250 docking models for each protein conformer in the provided ensemble (*w_vdw = 0*, *tolerance = 20*). Note that, during this stage, both the orientation and the position of the ligand are randomized before docking (randorien and rigidtrans enabled, ntrials = 5), with only the receptor held fixed at the origin, so that the crystallographic placement of the ligand relative to the receptor is not retained as a starting point. The ligand is, however, kept fully rigid at every stage (nseg2 = 0 in both refinement modules), and the ambiguous interaction restraints derived from the experimental binding site are active at all three stages, with no random restraint removal (randremoval = false). The top 5050 models were retained by HADDOCK score (equivalent to 50 per conformer on average, though selected globally rather than per conformer) and passed to semiflexible refinement, in which the side chains of selected residues (namely, those lining the binding site) were allowed to be flexible, while the remainder of the receptor and the ligand was kept rigid. The refined models were then subjected to structural optimization (*emref*) to further resolve possible strains remaining from the previous steps and improve HADDOCK scores. Poses were evaluated against the crystallographic reference structure at each stage (CAPRI evaluation, i-RMSD cutoff to identify interface residues set to 3.5 Å). Per-pose scores and the fraction of native contacts (*F*_nat_) were finally recorded after the *emref* stage. The complete HADDOCK3 workflow, with module parameters, is summarized in Table S5.

Subsequent *ad hoc* pose analysis was performed by calculating symmetry-aware RMSD values (via *symmrmsd* in *cpptraj*) using dual-reference ligand geometries to resolve apparent ligand-flipping issues.

As an internal benchmark on the present ligand set, the following additional calculations were also performed, using the same docking protocol described above: (i) single-template controls: the HM_4AKE(apo)_ and HM_1AKE(holo)_ model sets defined above were docked in place of the HM_gEDES_ ensemble; (ii) redocking control: each ligand was docked back into its own experimental holo receptor (we collectively label these structures HOLO_expt_), from which the ligand had been removed. This is the upper bound of the workflow and defines the noise floor of the RMSD metrics under our protocol.

#### Pose Evaluation

All refined poses were assembled into a multiframe trajectory file, with an accompanying index mapping each frame to its corresponding pose. For every pose, four RMSD metrics were computed relative to the experimental holo reference using *cpptraj* (AmberTools24):^45^ (i) Whole-protein RMSD (RMSD_prot_^α^), calculated on C_α_ atoms after global best-fit superposition; (ii) Binding-site RMSD (RMSD_BS_), heavy atoms of the binding-site residues, computed via *symmrmsd* to account for topological symmetry; (iii) Ligand RMSD (RMSD_lig_), ligand heavy atoms, evaluated without superposition (nofit) after the complex had been best-fit onto the binding-site heavy atoms; (iv) Binding-Mode RMSD (RMSD_BM_), combined heavy atoms of both the binding-site and ligand heavy atoms after collective best-fit superposition.

#### Conformational Clustering

Poses were clustered using the hierarchical agglomerative algorithm in *cpptraj*. Each pose was first superposed on the heavy atoms of the binding site and of the ligand. Next, pairwise distances were calculated with clusters merged until the minimum intercluster distance exceeded a 1.5 Å cutoff (ε = 1.5). Finally, the resulting cluster assignments, representative structures, and per-cluster member ensembles were saved.

#### Score Integration and Cluster Ranking

RMSD_BM/lig/BS/prot_ values were computed with *cpptraj* and merged with the HADDOCK3 output into a single per-pose dataset, which was the input for cluster ranking described below. Clusters were characterized and ranked using *in-house* Python scripts. For each cluster, we recorded its population, the mean of its three best (most negative) HADDOCK scores, the within-cluster distribution (mean ± standard deviation and range) of each RMSD metric and of the HADDOCK score, and the metrics of its representative pose, defined as the member with the lowest combined “binding-site + ligand” RMSD (RMSD_BM_). Clusters were ranked by their mean of top three HADDOCK score. We refer to the presence of a native-like pose anywhere in the docking output as ensemble recovery, and to its presence within the top 1-, 5-, or 10-ranked clusters as top-*N* pose prediction. All correlations reported below are Spearman rank coefficients (ρ) with two-sided *p*-values, chosen because the predictor-outcome relationships are neither linear nor Gaussian.

## Results and Discussion

In this section, we discuss the results obtained using gEDES-HM in terms of efficiency in transferring functional conformational ensembles across protein homologues, focusing on the reproduction of bound-like structures of the binding site and full proteins. Then, we discuss the relevance of these conformational ensembles in cross-docking calculations performed with HADDOCK3.

We first characterized the gEDES ensemble along established collective coordinates of the ADK transition, thereby defining the conformational features evaluated to assess the transferability of biologically relevant motions. In Figure 2, we report the projection of the conformational ensemble of *E. coli* ADK (namely the 101 structures extracted from the gEDES simulation described in ref 19) on the planes defined by the pairs of collective coordinates (*d*_1_, *d*_2_) and (θ_1_, θ_2_), together with the experimental references and the homologue structures. Note that 6 out of 22 homologues are “short-form” targets lacking 63–72% of the LID domain; as a result, for these systems, *d*_1_ and θ_1_ were computed over a partial selection of their residue ranges, namely 28–37% and 12–21% for the former and the latter, respectively. In the experimental holo structures of these six targets (Figure 2), the corresponding fractions are instead 28–37% and 6–18%: the *d*_1_ selection is identical, whereas the θ_1_ one is slightly smaller because some residues of the surviving LID segment are unresolved in those crystal structures while the homology models (Figures S2 and S3) carry them.

The gEDES representatives span 17.5 to 32.0 Å along *d*_1_ and 17.5 to 22.1 Å along *d*_2_, respectively, thus covering the whole interval between the open (4AKE, 29.6 and 21.8 Å) and the closed (1AKE, 20.5 and 18.3 Å) references. Note that the latter values agree with the published ones (29.5, 21.0) Å and (20.5, 18.3)^50^ to within 0.1 Å for *d*_1_ at both end points and for *d*_2_ at the 1AKE end point, and to within 0.8 Å for *d*_2_ at the 4AKE end point. Importantly, *d*_1_ and *d*_2_ are uncorrelated across the distribution (Spearman ρ = −0.007, *p* = 0.94), indicating that the ensemble spans a broad conformational space where the LID and NMP domains are not strictly coupled (as expected by construction in gEDES simulations).

Similar results were found along the (θ_1_, θ_2_) coordinates, which span 55.9 to 120.9° and 28.0 to 67.1°, respectively, thus bracketing again both 4AKE (94.8°, 61.5°) and 1AKE (62.8°, 27.0°). These end point values agree to within 1° with the approximate ones reported by Li et al.^51^ (about 95° and 61° for 4AKE, 68° and 28° for 1AKE), except for θ_1_ of 1AKE, which is about 5° smaller. We highlight that gEDES biases the shape and the volume of the pocket and not the above coordinates, and the 101 representatives are selected by cluster analysis for structural diversity rather than by statistical weight, so the ensemble is enriched in semiclosed (LID closed, NMP open) and closed (ligand-competent) states. The resulting distribution is therefore a biased, selection-filtered one and must not be read as a population or as a free-energy surface. Despite this limitation, it is intriguing to note that the generated ensembles reflect the proposed kinetics of the bound enzyme, whereby the LID closes before the NMP domain.^59^

To assess whether relevant intermediate states are included in the pool of gEDES conformations, we refer to the work by Li et al.,^51^ who divided the apo-to-holo transition in ADK among an open basin α, a semiopen–semiclosed basin γ, and a closed basin ζ based on the (θ_1_, θ_2_) coordinates. Importantly, the gEDES ensemble occupies all three regions, with 22% of the representatives in ζ, 21% in γ, 4% in α, and the remaining 54% in different subregions of the graph. Moreover, every one of the seven aforementioned structures is matched by a gEDES conformation to within 2.4°. Summarizing, the gEDES ensemble samples quite thoroughly the spaces of collective coordinates along the open-to-close ADK transition and includes conformations that resemble the putative intermediates.

To assess how much these properties transfer to each homologous protein in the pool, we projected the transferred ensembles HM_gEDES_ on the same sets of coordinates (Figures S2, S3). Almost all homologues lie inside the range spanned by the corresponding gEDES-HM ensemble onto the (*d*_1_, *d*_2_) or (θ_1_, θ_2_) coordinates, most of them being clustered around the closed reference structure, and at a median distance (angle) of 0.16 Å (2.4°) from the nearest model (Figures S2, S3, Table S4). The HM_gEDES_ ensembles behave very similarly to the original gEDES pool, with two informative exceptions.

The first is system 11 (2AK3), which is an AMP-only complex, with no ligand bound at the LID-CORE interface, thus corresponding to a semiclosed conformation. This is the only structure of the set that is not reached within 10° by any of the HM_gEDES_ conformations. However, also for this system, we were able to generate almost 14% of conformations reproducing the bound-like conformation of the AMP binding site (Table S2). The second is system 14, which lies between γ and α. With respect to this structure, the HM_gEDES_ ensemble includes a conformation distant only ∼3° in the (θ_1_, θ_2_) coordinates; however, we were unable to recover a bound-like conformation of the GP5 binding site.

These results further highlight the lack of correlation between reproducing the collective motions of ADK and reproducing the fine details of the competent binding-site geometry (enabling the recovery of native-like binding modes in docking calculations; see below).

Next, we focus on the evaluation of the ability of our protocol to recover, among the transferred ensembles of conformations, structures that are similar to the bound protein and/or binding site. Native-like binding-site conformations (RMSD_BS_ ≤ 2.5 Å) were obtained for 22, 2, and 24 out of 26 complexes (corresponding to 84.6%, 7.7%, and 92.3%, respectively) using HM_gEDES_, HM_4AKE(apo)_, and HM_1AKE(holo)_, respectively (Figure 3 and Table S2). When considering the full protein, these numbers become 18, 0, and 19 out of 22, corresponding to 81.8%, 0%, and 86.4% of the targets (Table S2). These results show that competent binding-site and whole-protein geometries can be recovered for most members of the family despite their sequence and conformational diversity. The challenging systems are 12 and 14, for which no native-like binding site was obtained, and system 11, whose binding site but not whole-protein geometry is recovered in ∼14% of the models, due to LID opening in the AMP-bound complex. The implications of the challenges encountered in reproducing the binding-competent structures of these targets for what concerns docking accuracy will be discussed in the next section.

**Figure 3:**
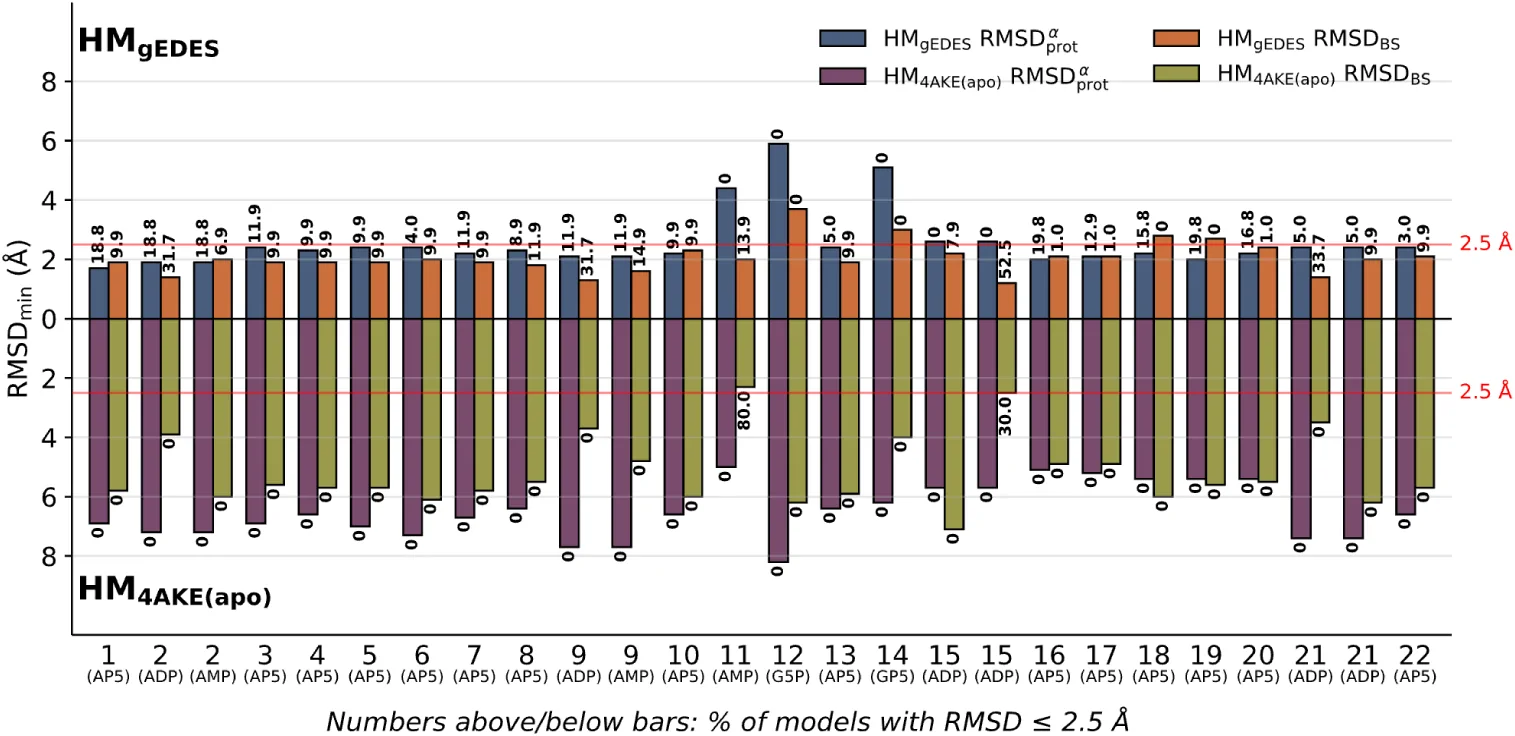
Structural accuracy with respect to the holo conformations of homology models built upon gEDES clusters and the single-template of apo experimental control. RMSD distributions of homology models generated using gEDES clusters (top) and the single experimental apo ADK template (PDB ID: 4AKE, bottom), each evaluated against the corresponding holo reference structures listed in Table 1. Whole-protein RMSD was calculated on C_α_ atoms (blue, purple), whereas binding-site RMSD_BS_ (orange, green) was calculated using heavy atoms. Bars represent the percentage of models with RMSD values ≤ 2.5 Å for the whole protein and BS, based on the HM_gEDES_ and HM_4AKE(apo)_ sets of conformations. The values shown below and above the bars indicate the proportion of structures falling below the defined cutoff. The corresponding co-crystal experimental ligands are indicated in parentheses on the *x*-axis. Overall, native-like binding-site conformations were identified for 22 out of 26 (84.6%) complexes when using the gEDES-derived templates, compared to 7.7% when using the single apo template HM_4AKE(apo)_. Additional details are provided in Table S2, and structural comparisons of the best models with the corresponding experimental references (including holo template) are shown in Figure S4. Additional comparison and results for the holo-template control HM_1AKE(holo)_ are reported in Table S2.

Finally, we evaluated whether the HM_gEDES_ conformational ensembles improve the *in silico* prediction of the corresponding protein-ligand complexes. For this purpose, we performed ensemble docking calculations with HADDOCK3 on the following sets: HM_gEDES_, HM_4AKE(apo)_, HM_1AKE(holo)_, and HOLO_expt_ (see Methods for details). The systems for which we recovered a native-like binding mode (RMSD_BM_ ≤ 2.5 Å) were, respectively, 24, 2, 24, and 25 (out of 26 complexes, corresponding to 92.3%, 7.7%, 92.3%, and 96.2% of the targets, respectively; see Figure 4 and Table S6). Table S6 reports also the values corresponding to the number of native-like poses in the top 1, 5, and 10. Notably, the percentage of native-like poses within the top 10 ones (80.8%) remains close to the success rate obtained at the modeling stage (84.6%), indicating again that docking exploits most of the binding-competent conformations generated in the modeling stage. Actually, the accuracy obtained in docking calculations using HM_gEDES_ is marginally higher than that recorded at the homology modeling stage (92.3% vs 84.6%): in fact, every target for which a sub-2.5 Å binding-site model was available also produced a native-like pose, and docking additionally recovered two targets (systems 18 and 19) whose best homology-model binding site lay just above this threshold. Furthermore, the performance of HM_gEDES_ is equivalent to that of HM_1AKE(holo)_ and slightly worse than that obtained by redocking the ligands onto the experimental structures of their corresponding receptors, although we must note that the accuracy of the recovered poses is on average lower in the first case (median RMSD_BM_ 2.1 Å against 1.1 Å for HM_1AKE(holo)_ and 0.6 Å for HOLO_expt_).

**Figure 4:**
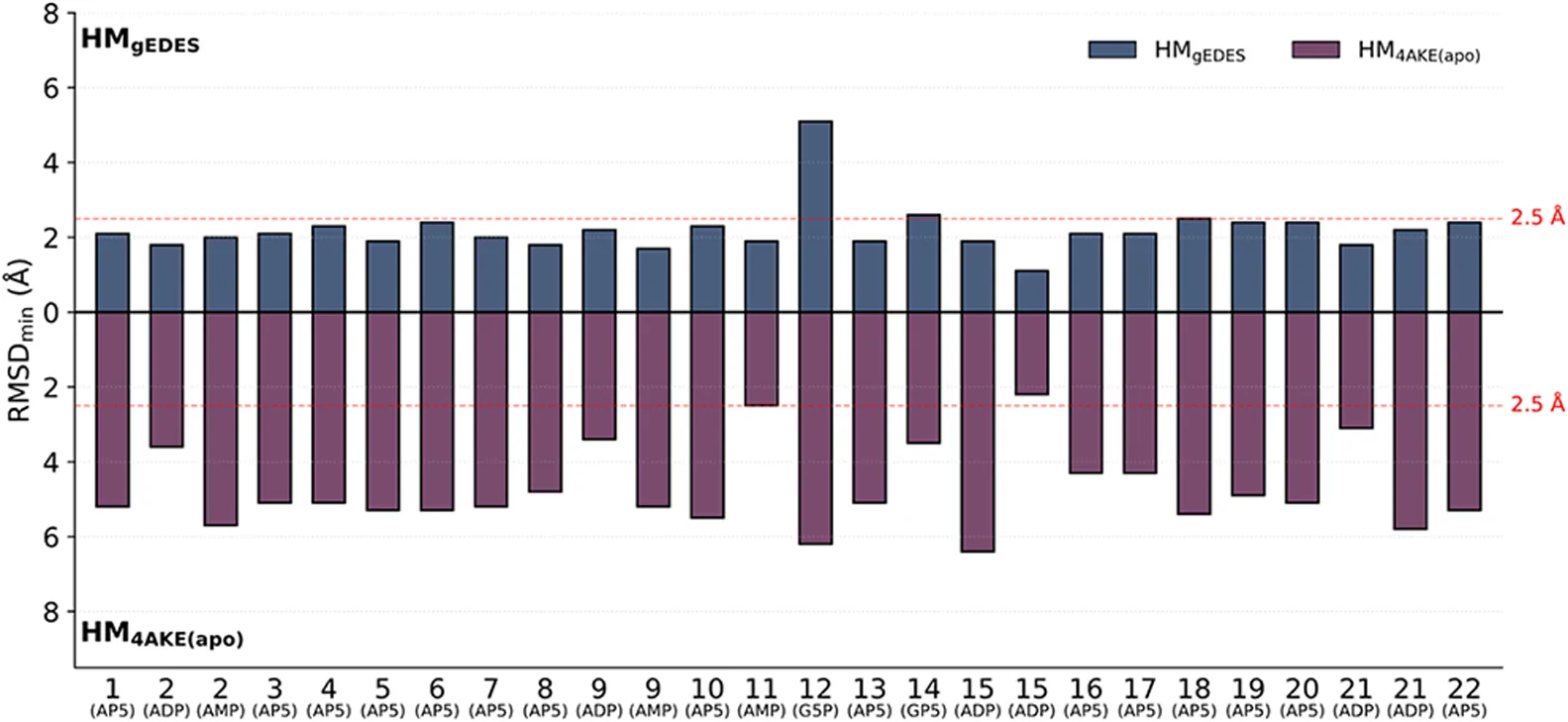
Native-like poses recovered by HADDOCK3 ensemble docking. The minimum RMSD values of native-like poses are reported for the binding mode (RMSD_BM_, [Å]; heavy atoms of both the binding site and the ligand) for homology models derived from gEDES clusters (top; HM_gEDES_) and the experimental apo template (bottom; HM_4AKE(apo)_). Structural alignment and RMSD_BM_ calculations were performed using the heavy atoms of the ligand and binding-site residues. The corresponding experimental ligands are indicated in the parentheses on the *x*-axis. Overall, native-like binding modes were recovered for 92.3% (7.7%) of the complexes when using the HM_gEDES_ (HM_4AKE(apo)_)-derived homology models. Top poses of each target are listed in Table S6, and structural comparisons with the experimental binding modes are shown in Figure S5. In addition, the results of the holo-template control HM_1AKE(holo)_ and of the redocking control HOLO_expt_ are reported in Table S6.

Nonetheless, the performance of HM_gEDES_ is relevant, since several prospective applications could not exploit prior structural information.

Interestingly, unrecovered complexes were again systems 12 and 14 when using the HM_1AKE(holo)_ ensemble and system 12 when using the HOLO_expt_ one (Table S6), indicating that failures cannot be attributed clearly to the use of transferred ensembles only. More generally, model and pose accuracy showed no strong dependence on the sequence identity of either the binding site or the full protein to the templates (Figure S6), indicating that gEDES-HM allows recovering native-like geometries across the family sequence diversity rather than only for close homologues. A conformer-level analysis of the docking results on each system (Table S7) reveals that the number of conformers yielding a native-like pose ranges from 1 to 45 out of 101, with a median of 7 over the 24 recovered complexes, confirming that our protocol can generate a low but still significant (and crucial for the purpose of reproducing binding poses) fraction of binding-competent conformations.^19,20^

### Analysis of Unsuccessful Cases and Limitations of Our Approach

In this section, we discuss possible reasons behind the failure of our protocol to accurately reproduce (independently of the ranking) the experimental binding modes of systems 12 and 14 (5.1 and 2.6 Å), as well as other limitations of gEDES-HM.

Analysis of challenging cases reveals distinct patterns, although both failures cannot be explained by sequence identity or by global model quality. Indeed, binding-mode accuracy showed no significant dependence on the binding-site sequence identity (Spearman ρ = −0.28, *p* = 0.167) or on the global parameter RMSD_prot_ (ρ = 0.15, *p* = 0.458) over the 26 complexes. For instance, systems 11 and 12 have both 68% binding-site identity to the template and both feature large backbone deviations from the corresponding experimental structures (Tables 1 and S2).

However, the HM_gEDES_ ensemble of system 11 contains a conformation with a significantly lower value of RMSD_BS_ from the binding-competent structure (2.0 Å vs 3.7 Å in system 12). As found in previous works,^19–21^ the fidelity in reproducing fine details of the binding site is key to recover native-like binding modes. In particular, the number of productive conformers per complex is strongly anticorrelated with the minimum RMSD_BS_ of the corresponding model ensemble (Spearman ρ = −0.86, *p* = 1.8 × 10^–8^ over the 26 complexes, and ρ = −0.82, *p* = 8.6 × 10^–7^ over the 24 recovered ones). While several factors can affect the outcome of docking calculations, it is worth noting that systems 12 and 14 display the largest RMSD_BS_ values relative to their corresponding reference geometries in the dataset (Table S2). Finally, we note that *F*_nat_ remained relatively high for both systems 12 and 14 (0.75 and 0.74, respectively), indicating that the ligand still occupies the correct (sub)­site and that the high RMSD_BM_ reflects physical distortion of the modeled pocket rather than mislocalization of the compounds.

In summary, HM_gEDES_ ensembles employed in (restrained) docking with HADDOCK3 are robust to wide backbone divergence and low global sequence identity down to 30%, i.e., the practical operational boundary of this workflow is set upstream by the fidelity of the binding-site model. Future work could address some drawbacks of the current protocol by sampling alternative conformations of the binding sites, for example, by expanding the template pool with gEDES simulations of additional (evolutionarily distant) representative family members or by introducing limited flexibility of the binding site and of the ligand into the docking protocol. More generally, further limitations of transferring a single-reference ensemble to homologous proteins should be mentioned:

- The protocol assumes that the conformational states relevant to every homologue are already represented in the reference ensemble. This might not be true if family members have evolved distinct functional states, altered kinetics, different oligomeric arrangements, or ligand-specific induced-fit responses, and it is consistent with the fact that both failures reported here belong to the AK3-type GTP:AMP phosphotransferases, that is to the subfamily whose triphosphate substrate differs from that of the reference enzyme.
- The conformational diversity sampled across the reference ensemble is transferred to family members only insofar as they possess the domains whose motion generates that diversity. The diversity of the present pool is overwhelmingly due to LID rather than NMP fluctuations (Δ*d*_1_ 14.5 Å vs Δ*d*_2_ 4.6 Å, see Figure 2), and the six systems (15 to 20) lacking between 63% and 72% of the LID domain are precisely those for which the fewest conformers are productive: every complex with two or fewer productive conformers is a short-form ADK, while no recovered long-form target has fewer than four.
- The gEDES simulation reused here was run on the apo enzyme in the absence of Mg^2+^, whereas the physiological reaction requires the ion. Bound-like binding-site conformations were nonetheless recovered for most of the family members even without the presence of the ion. These findings are in line with previous results highlighting the small influence of Mg^2+^ on the stability of open and closed ADK conformations,^40,41^ in contrast with its larger impact on the enthalpic barrier and the transition-state ensemble.^31^ The inclusion of Mg^2+^ (and more generally, additional cofactors) in gEDES simulations will be addressed in follow-up projects.
- Finally, for the complexes in which only one or two conformers are productive, success rests on those conformers surviving clustering, comparative modeling, and DOPE-based selection, which is a fragility of the current selection step rather than of the sampling.

## Concluding Remarks

We have presented a scalable computational framework enabling the transfer of wide conformational ensembles of a single protein to an entire homologous family, drastically reducing the computational costs of generating druggable conformations for families of proteins. Applied to 26 protein-ligand complexes from the adenylate kinase family, the approach reproduced native-like binding modes for ∼92% of the complexes, approaching the performance obtained by redocking into the corresponding experimental holo receptors (structural information that would not be available in a prospective application). These conclusions are established within one protein family, whose dominant conformational change is a well-characterized domain closure, and their extension to families with different functional motions remains to be demonstrated. Nonetheless, we anticipate that similar results to those presented here for ADK could be reproduced for other protein families as far as the similarity of the putative binding site(s) is high enough to reproduce its bound-like structure with good accuracy. The property is valuable for orphan and novel ADK targets for which close structural templates are unavailable, because sampling of conformational space can compensate for global template uncertainty as long as the binding-site conformation is represented in the ensemble. It also shifts the bottleneck from conformational sampling to conformer selection, since a global statistical potential such as DOPE can discard models whose binding-site geometry is closest to the target. In addition, while our docking strategy requires some knowledge of the binding site(s), which here relies on the holo reference structures, this is not intrinsic to the method. Indeed, pockets can be detected directly on the gEDES conformers, as we showed previously for ADK,^19^ allowing the pipeline to be run in the absence of any holo structure of the target.

Future versions of the workflow could therefore combine global model quality with local criteria, such as pocket geometry, side-chain packing, and binding-site stereochemistry, in a pocket-aware or multicriteria selection score. In addition, possible applications actively pursued in our lab (which will be the subject of future works) include virtual screening and structure-based hit identification for family members that lack experimental structures and are known only through their sequences; prioritization of cryptic or allosteric pockets absent in the available crystal structures; and cross-member selectivity assessment from a single simulated ensemble.

## Supplementary Material



## Data Availability

All scripts employed to generate homology models, to perform docking calculations, and to perform post-processing are available on Zenodo (https://zenodo.org/records/21534586). At the latter link, we also uploaded all the homology-modeled structures of all proteins, and the best complex structures predicted using our workflow.

## Notes

Multiple sequence alignment of the 22 adenylate kinase homologues (Figure S1); conformational ensemble transferred to each homologue on the two center-of-mass distances of the ADK transition (Figure S2); the same ensembles on the two interdomain angles (Figure S3); comparison of the experimental structures with the best gEDES-derived homology models (Figure S4); comparison of the experimental binding modes with the best HADDOCK3 poses (Figure S5); model and pose accuracy against binding-site and protein sequence identity (Figure S6); binding-site sequence identity of each homologue to the reference adenylate kinase (Table S1); RMSD of the homology models to the holo reference structures (Table S2); projection of the reference, representative, homologue, and intermediate structures onto the collective coordinates (Table S3); closest member of the transferred pool for each long-form homologue (Table S4); HADDOCK3 ensemble cross-docking workflow and module parameters (Table S5); native-like poses identified by HADDOCK3 under the four receptor conditions (Table S6); number of productive conformers per complex (Table S7) (PDF)
