# Supplementary material for: Mapping Functional Transitions across Protein Homologues from a Single Simulation: Application of gEDES-HM to Adenylate Kinases
Source: J Chem Inf Model. 2026 Oct 5;66(20):13586–96. doi: 10.1021/acs.jcim.6c02521 (PMC13715475; doi:10.1021/acs.jcim.6c02521)
Supplement: Supplementary file 1 [file ci6c02521_si_001.pdf]

## Supplementary Information for

# Mapping functional transitions across protein homologues from a single simulation: application of gEDES-HM to adenylate kinases

Han Kurt[1], Mohd Athar[1], Andrea Basciu[2,*], Victor Reys[3], Alexandre M.J.J. Bonvin[3], Attilio V. Vargiu[1,*]

1. Physics Department, University of Cagliari, Cittadella Universitaria, Monserrato (CA) I-09042, Italy
2. WAY4WARD, Via Ruggero Bonghi 11/B, 00184 Roma, Italy
3. Bijvoet Centre for Biomolecular Research, Faculty of Science - Chemistry, Utrecht University, Padualaan 8, Utrecht 3584 CH, The Netherlands

* Address correspondence to: a.basciu2@gmail.com or avargiu@unica.it

## Supplementary Figures

**Figure S1.** Alignment of the 22 adenylate kinase sequences used in this study, aligned with Clustal Omega (v1.2.4)[1], reformatted with MView[2], and rendered with ESPript[3]. Sequences are labelled by their internal identifier and UniProt[4] accession (PDB ID for systems 21 and 22), with E. coli adenylate kinase (REFERENCE-ADK | P69441) as the reference on the top line; residue numbering along the top refers to this reference sequence. Strictly conserved positions (identical in all sequences) are shown as white letters on a red background, positions conserved in physicochemical character are boxed and shown in red, and non-conserved residues are in black. Dots denote gaps introduced by the alignment. This figure gives the global view of sequence divergence across the full-length proteins; the same alignment restricted to the 25 binding-site positions is tabulated in Table S1.

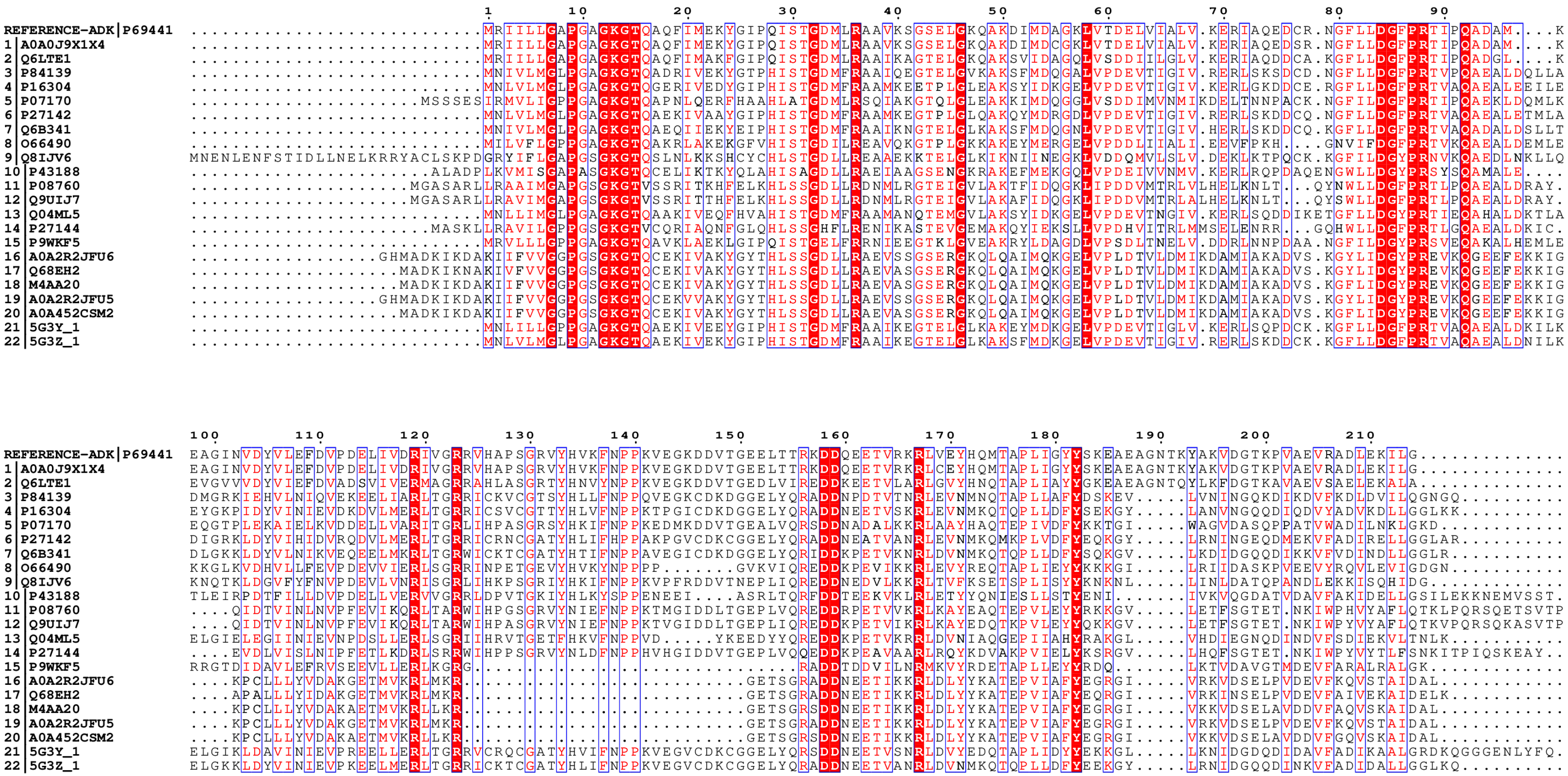

**Figure S2. Conformational ensemble transferred to each adenylate kinase homolog, on the two center-of-mass distances of Figure 2A.** One panel per target, in the order of Table 1, labeled with reference PDBID, ligand, and sequence identity. Grey, the 101 gEDES representatives of E. coli ADK (the transferred pool, identical in every panel); colored, the 101 homology models of that target, one per representative, blue for long-form, orange for the six short-form targets (15 to 20), red for systems 12 and 14, where no native-like structures were recovered. Open square and diamond, the E. coli references (4AKE open apo, 1AKE closed holo); filled triangle, the experimental holo structure of the target. Δ is the in-plane distance from that structure to the nearest of its 101 models, replaced for the short-form targets by $\Delta_2$, the same minimum on $d_2$ alone.

Every ensemble reproduces the amplitude of the parent pool, spanning 13.6-14.7 Å along $d_1$ (14.5 Å for the pool) and 4.4-5.3 Å along $d_2$ (4.6 Å). Models reproduce the coordinates of their parent representative to a median of 0.44 Å in $d_1$ over the sixteen long-form targets and 0.15 Å in $d_2$ over all 22. The experimental holo structure of every target lies within its ensemble, with a median Δ of 0.16 Å and a maximum of 1.55 Å for system 11 (2AK3), the AMP-only complex, whose LID has no reason to close. In the six short-form targets only 28-37% of LID residues are present, so $d_1$ is computed over a partial domain and is not comparable across panels; these panels are shaded and labeled with that fraction.

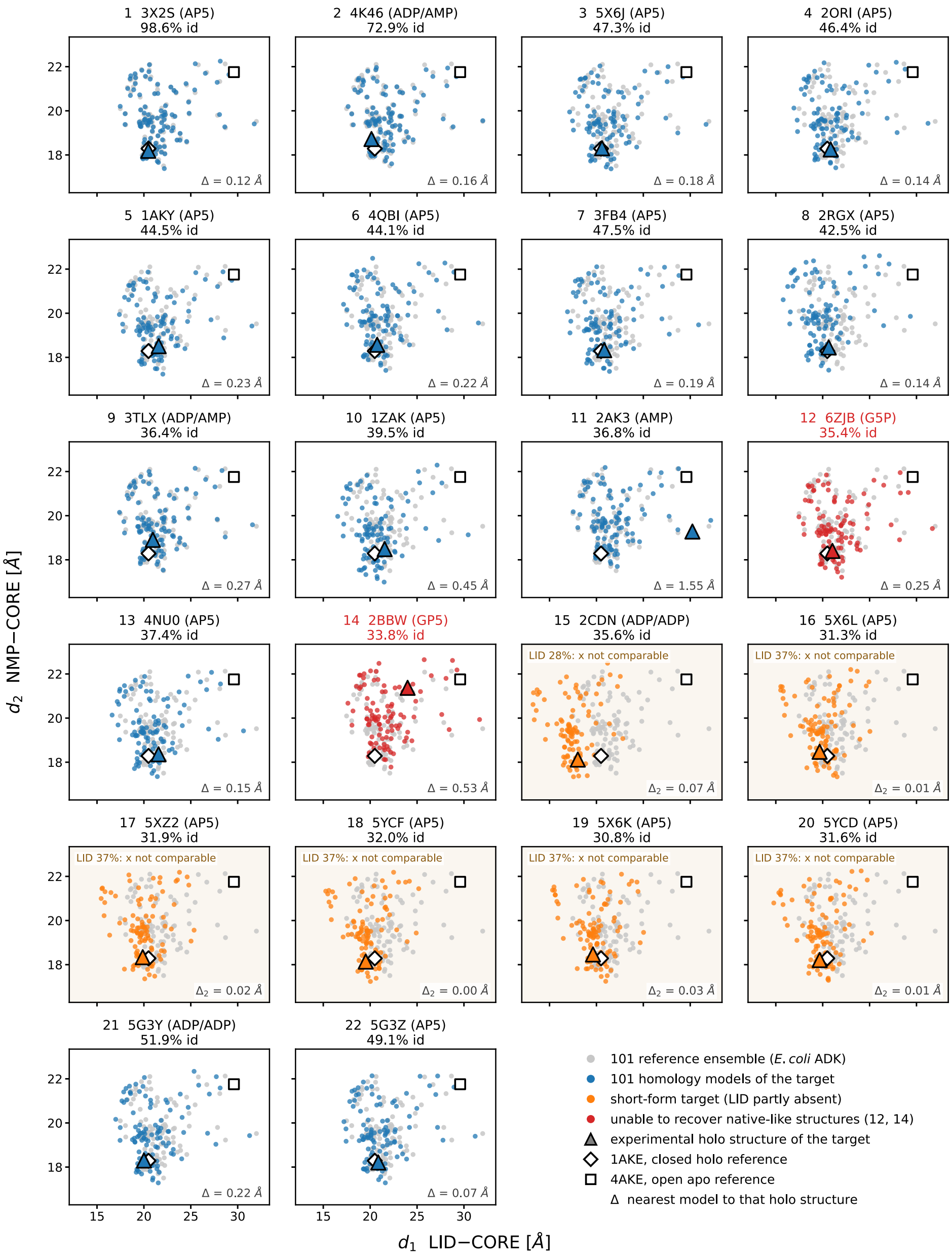

**Figure S3. Conformational ensemble transferred to each adenylate kinase homolog, on the two interdomain angles of Figure 2B ($\theta_1$ LID-CORE, $\theta_2$ NMP-CORE), with symbols, colors, and Δ as in Figure S2.** Every ensemble reproduces the amplitude of the parent pool, spanning 62.3-66.8° along $\theta_1$ (65.0° for the pool) and 38.5-40.9° along $\theta_2$ (39.1°), the short-form panels running wider in $\theta_2$ (up to 47.6°). Models reproduce the coordinates of their parent representative to a median of 1.87° in $\theta_1$ over the sixteen long-form targets and 1.54° in $\theta_2$ over all 22. The experimental holo structure of every target lies within its ensemble, with a median Δ of 2.35° and a maximum of 15.83° for system 11 (2AK3). In the six short-form targets only 12-21% of the LID residues used for $\theta_1$ are present, so $\theta_1$ is not comparable across panels; these panels are shaded and labeled with that fraction.

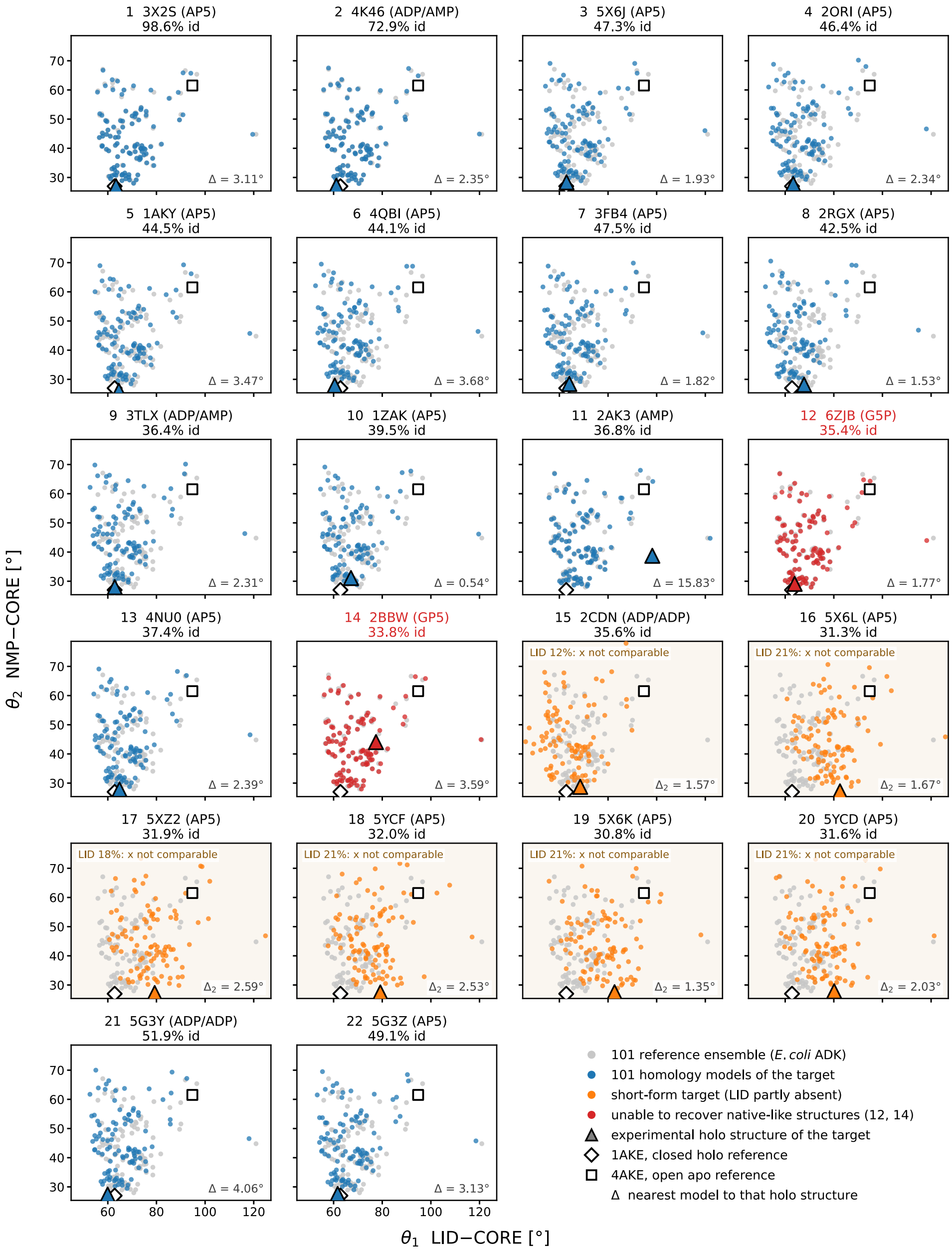

**Figure S4. Comparison between the experimental structures used in this work and the best homology models obtained for the same proteins using the gEDES clusters as templates.** Throughout, the experimental reference and homology model structures are shown in grey and green, respectively. The first column reports the system number followed by the reference PDB ID and the relevant ligand (in parentheses). In the middle column, each homology model is superimposed on its experimental reference structure, along with the value of the $RMSD_{prot}^{\alpha}$ between them. Transparent surfaces represent the molecular envelope of the ligands, colored hot-pink (AP5), blue (ADP), orange (AMP), yellow (GP5), and yellow-orange (G5P). The binding site of the same complex shown in the right column, along with the heavy-atom $RMSD_{BS}$. For clarity, only sidechain atoms of binding site residues are shown, and $C_{\alpha}$ atoms of homology models are colored along a rainbow gradient.

| System | Protein | Binding site |
|---|---|---|
| 1<br>ref: 3x2s (AP5) | 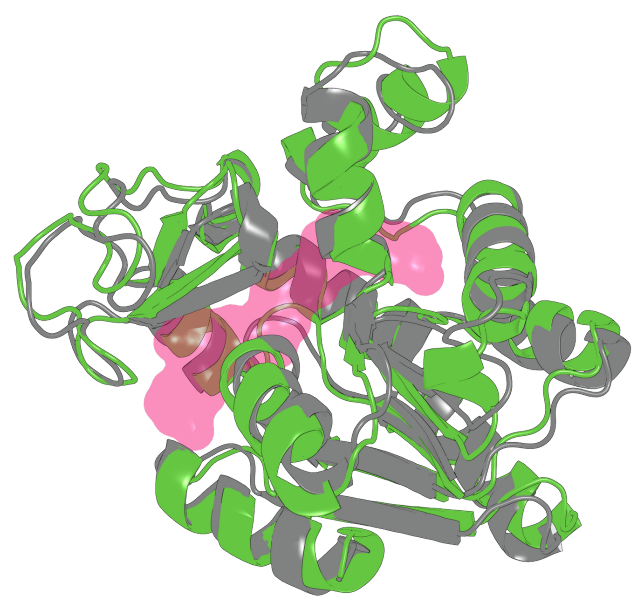 | 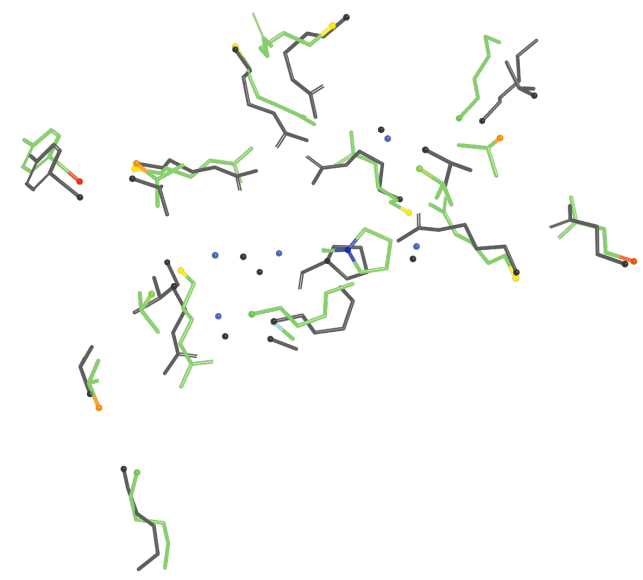 |
| | 1.7 Å | 2.0 Å |
| 2<br>ref: 4k46 (ADP) | 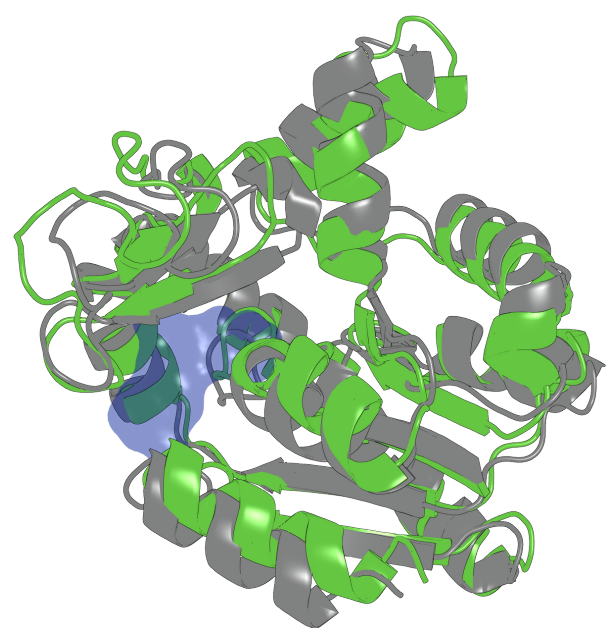 | 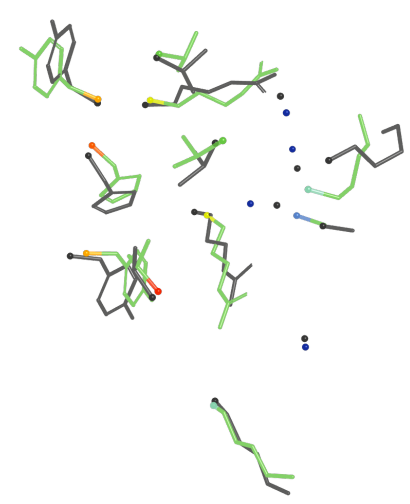 |
| | 2.1 Å | 2.0 Å |
| 2<br>ref: 4k46 (AMP) | 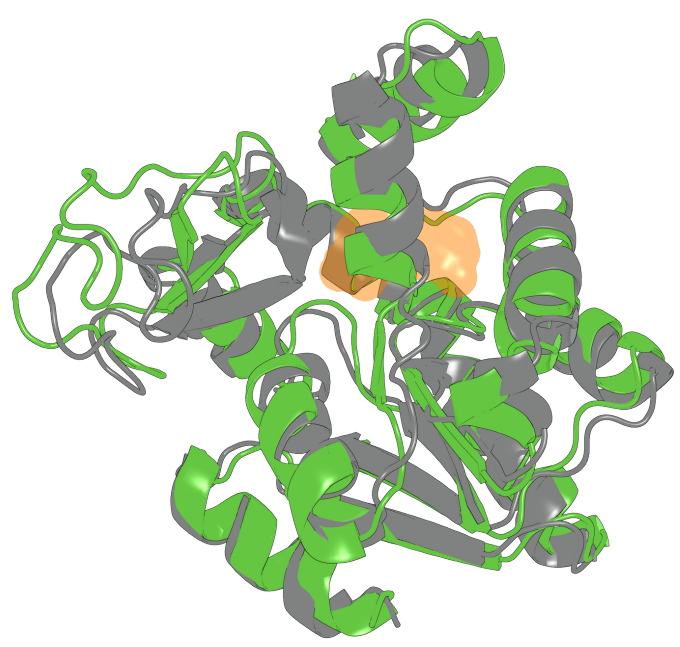 | 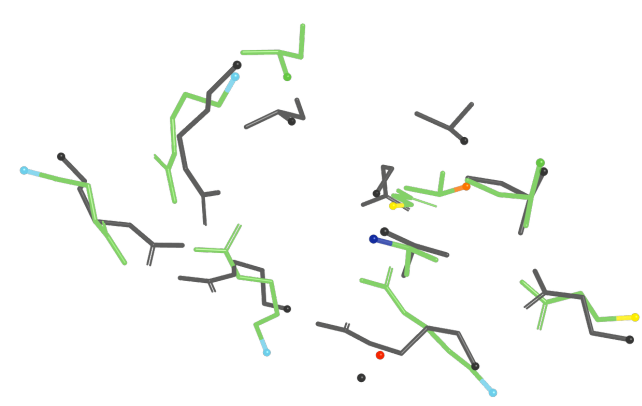 |
| | 2.1 Å | 2.0 Å |

| | | |
|---|---|---|
| 3<br>ref: 5x6j (AP5) | 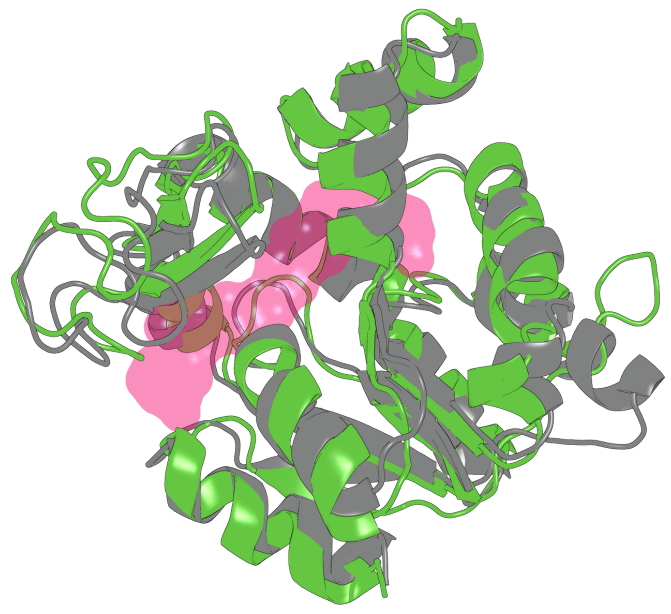 | 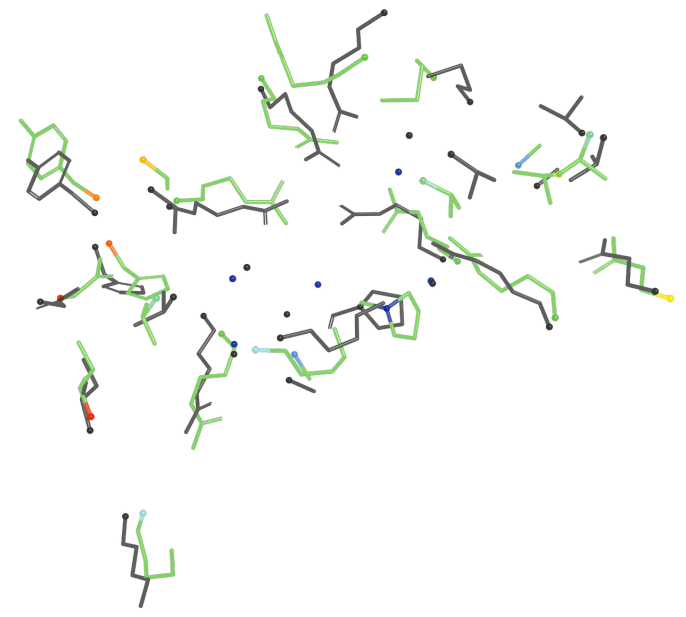 |
| | 2.4 Å | 1.9 Å |
| 4<br>ref: 2ori (AP5) | 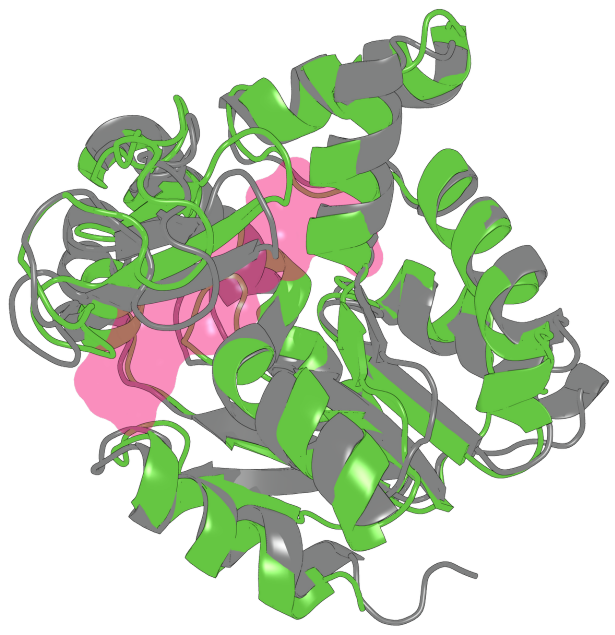 | 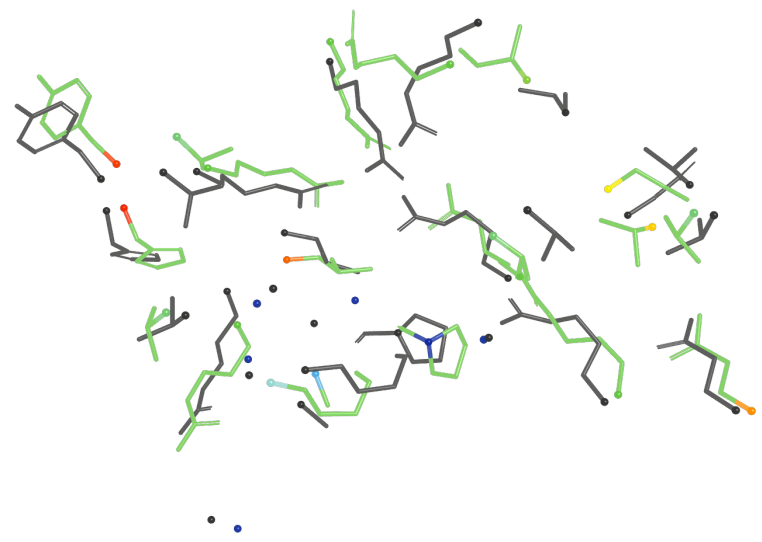 |
| | 2.3 Å | 1.9 Å |
| 5<br>ref: 1aky (AP5) | 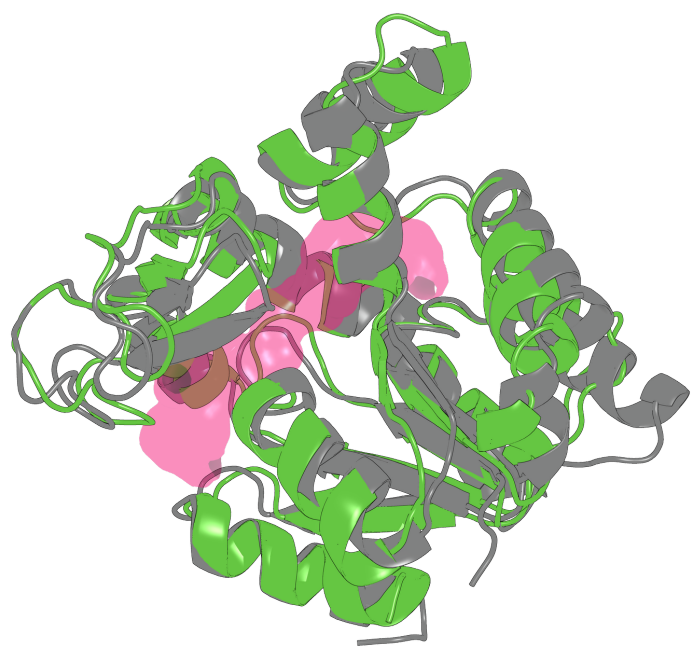 | 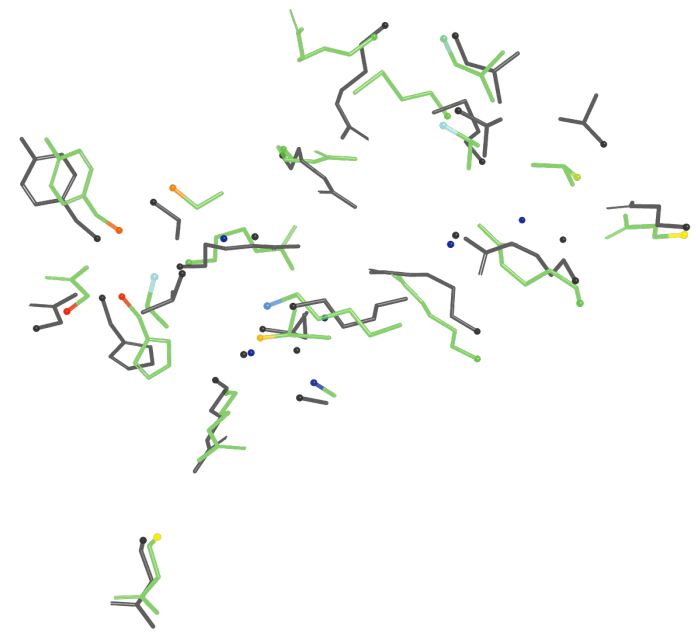 |
| | 2.4 Å | 1.9 Å |
| 6<br>ref: 4qbi (AP5) | 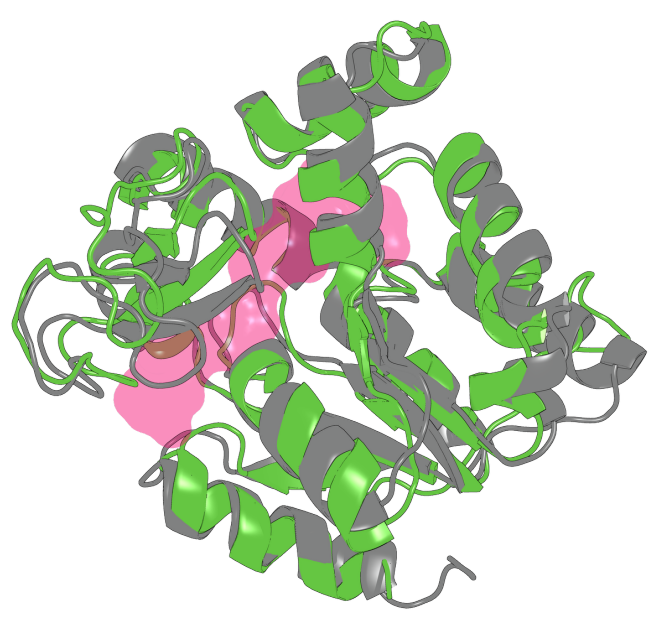 | 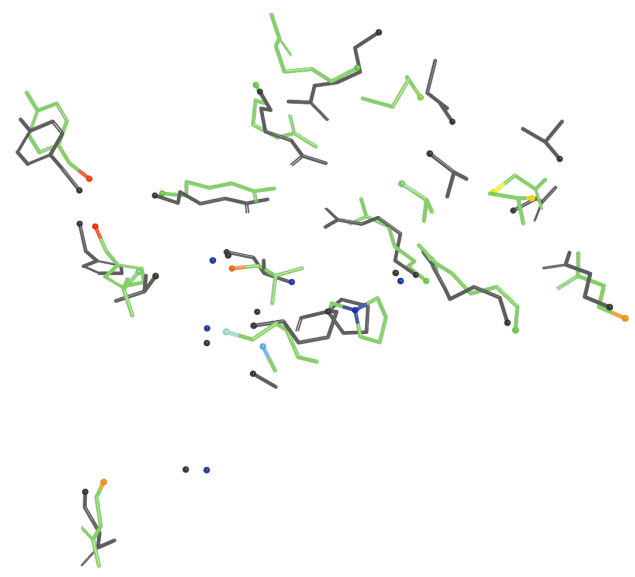 |
| | 2.5 Å | 2.0 Å |

| | | |
|---|---|---|
| 7<br>ref: 3fb4 (AP5) | 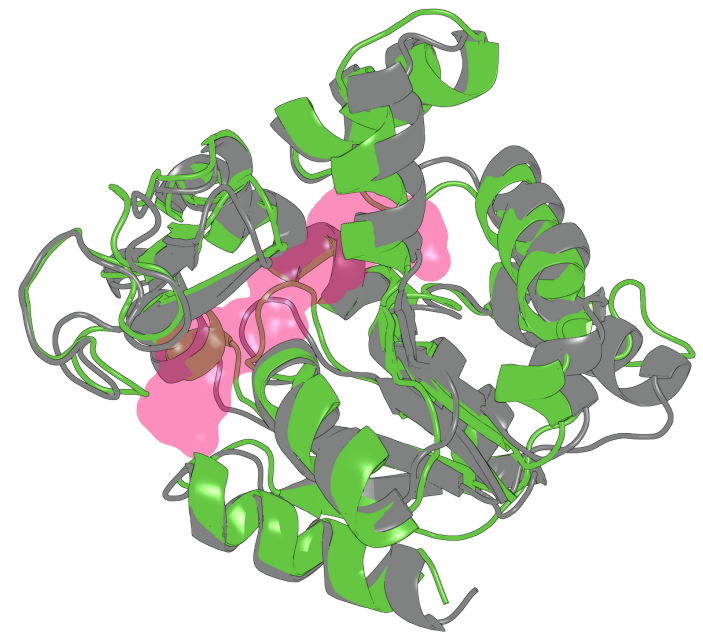 | 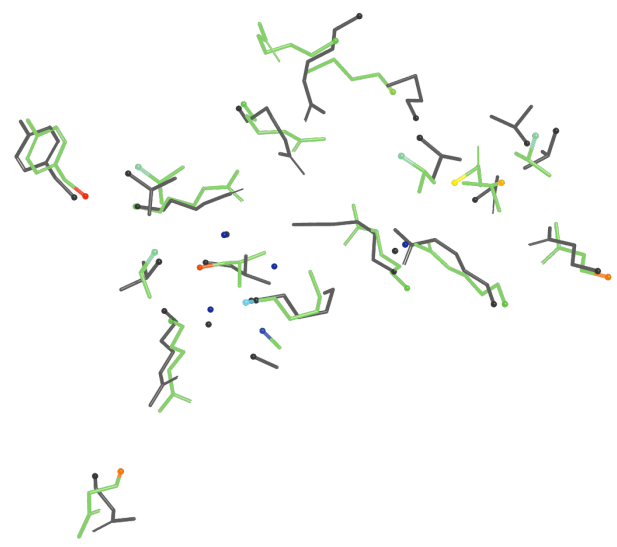 |
| | 2.3 Å | 1.9 Å |
| 8<br>ref: 2rgx (AP5) | 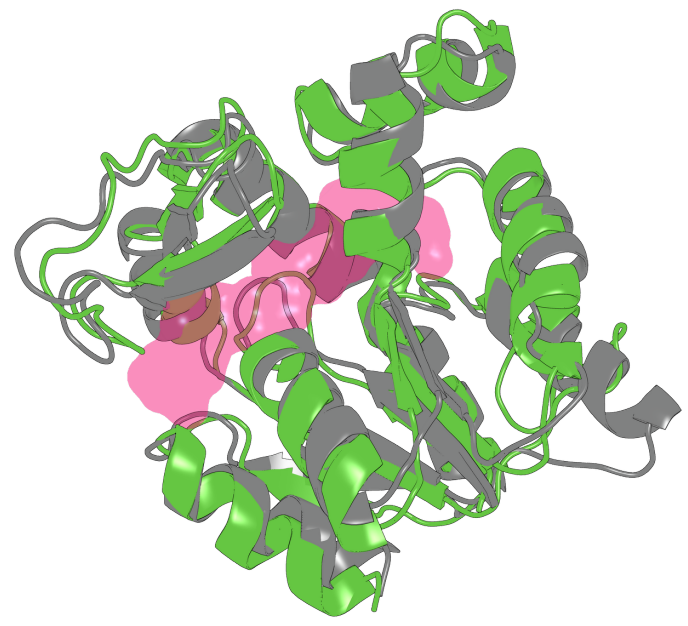 | 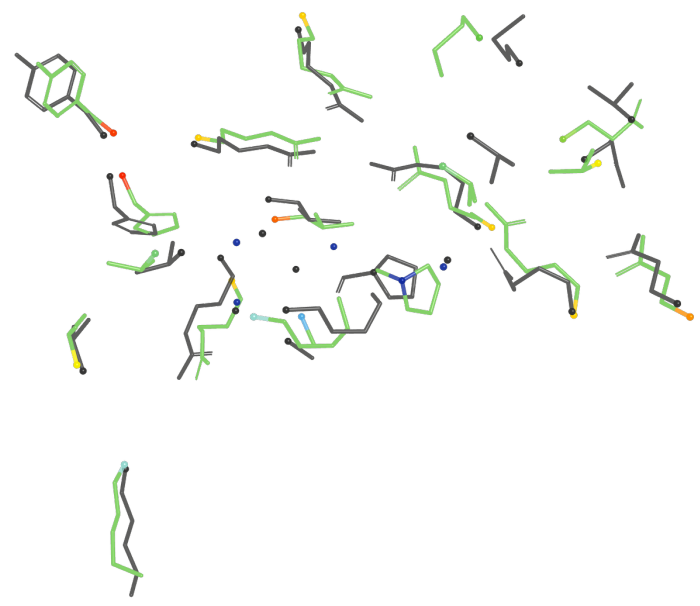 |
| | 2.4 Å | 1.8 Å |
| 9<br>ref: 3tlx (ADP) | 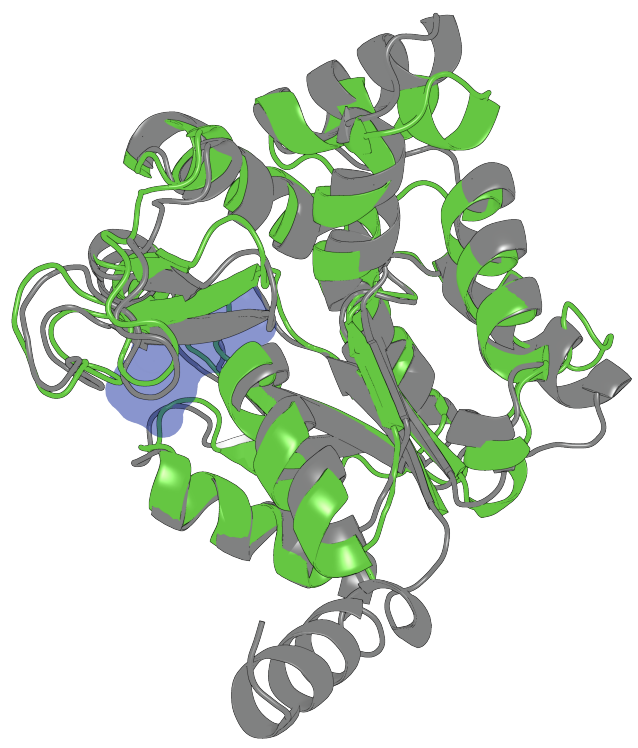 | 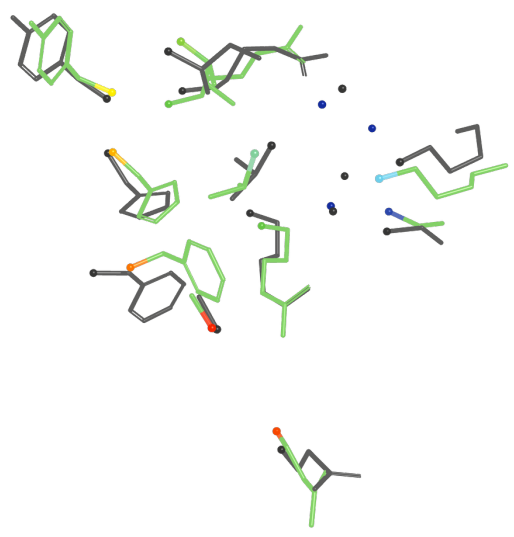 |
| | 2.4 Å | 1.9 Å |
| 9<br>ref: 3tlx (AMP) | 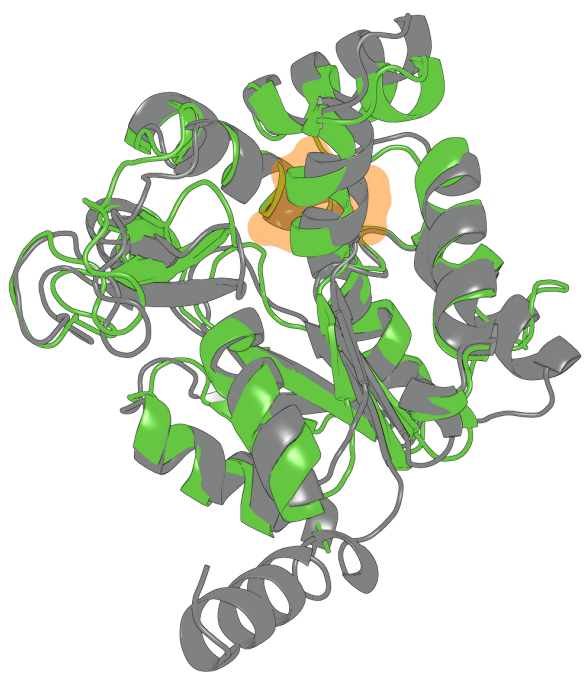 | 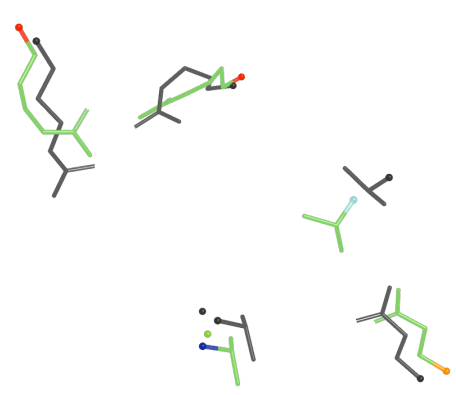 |
| | 2.3 Å | 1.7 Å |

| | | |
|---|---|---|
| 10<br>ref: 1zak (AP5) | 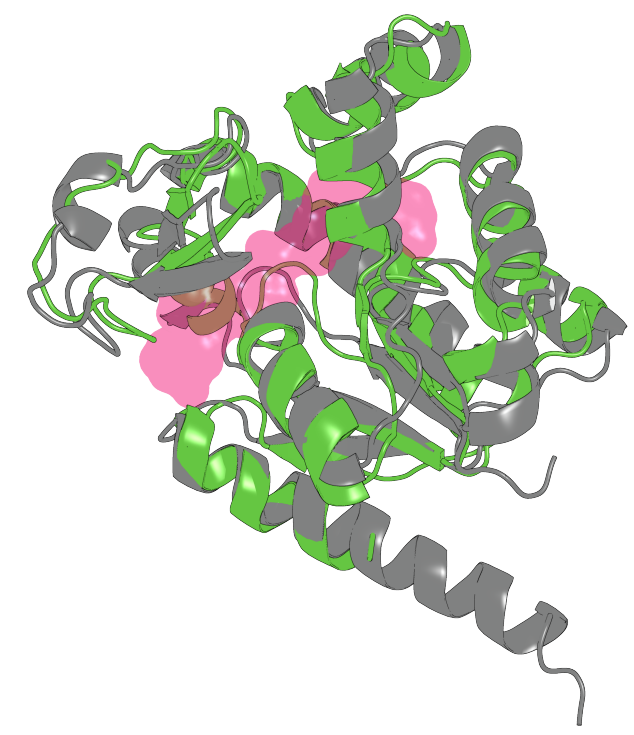 | 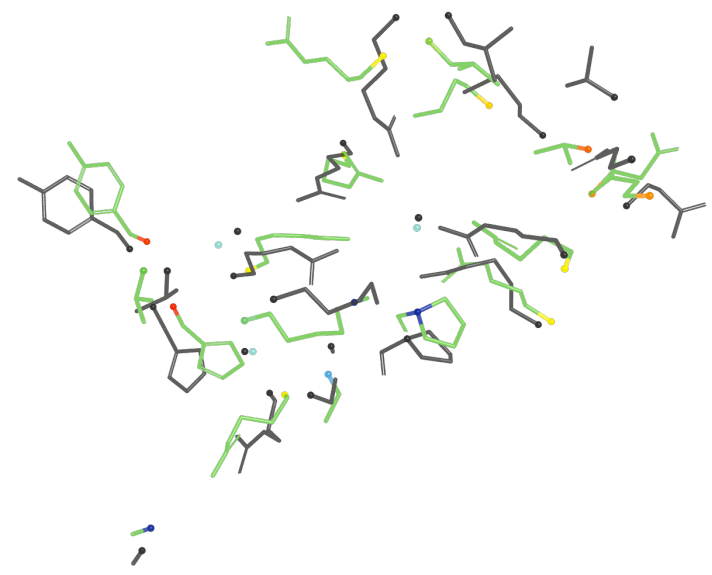 |
| | 2.3 Å | 2.3 Å |
| 11<br>ref: 2ak3 (AMP) | 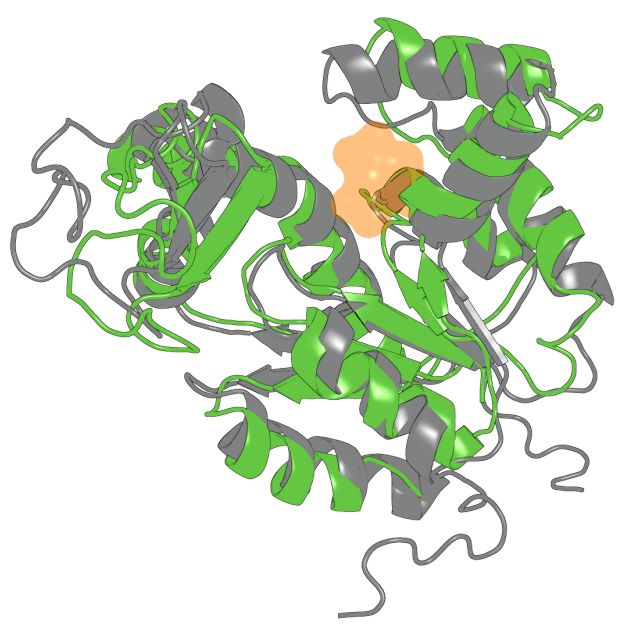 | 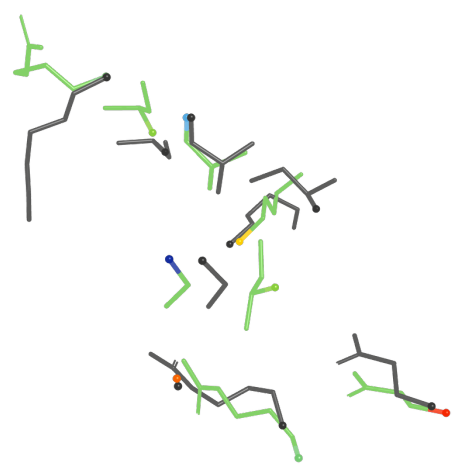 |
| | 4.6 Å | 2.5 Å |
| 12<br>ref: 6zjb (G5P) | 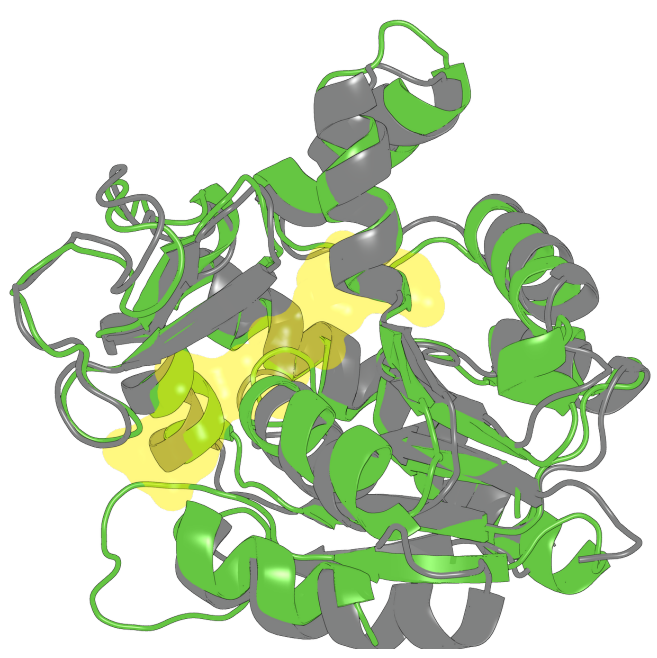 | 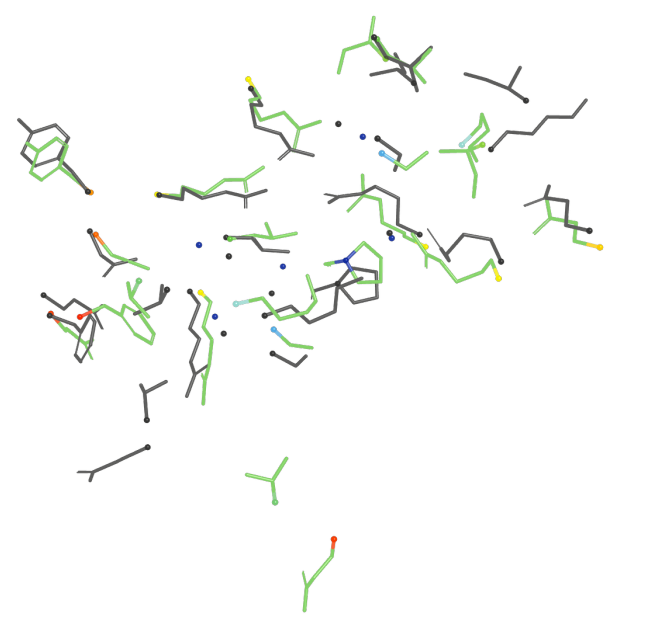 |
| | 6.1 Å | 3.7 Å |
| 13<br>ref: 4nu0 (AP5) | 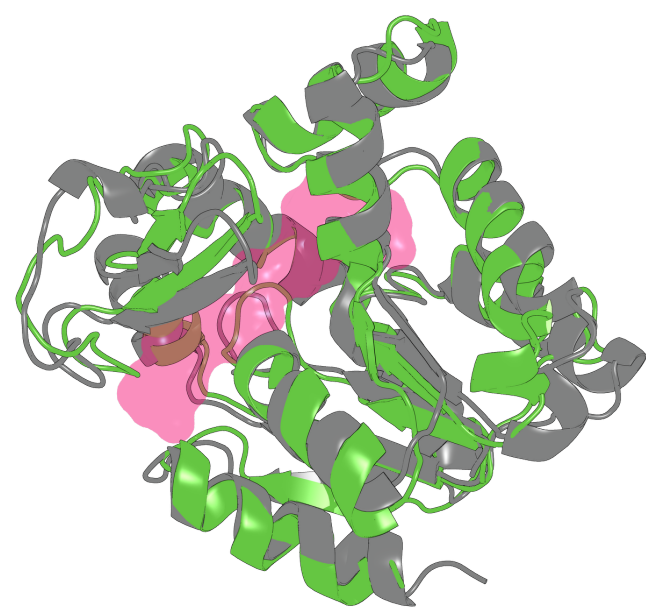 | 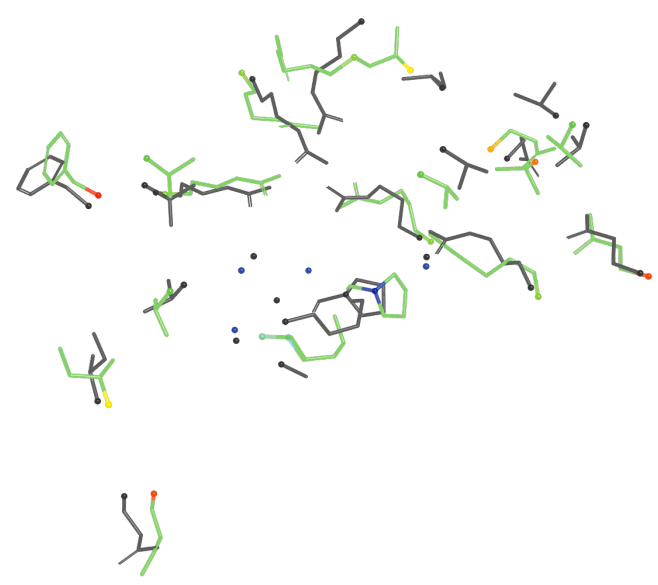 |
| | 2.4 Å | 1.9 Å |

| | | |
|---|---|---|
| 14<br>ref: 2bbw (GP5) | 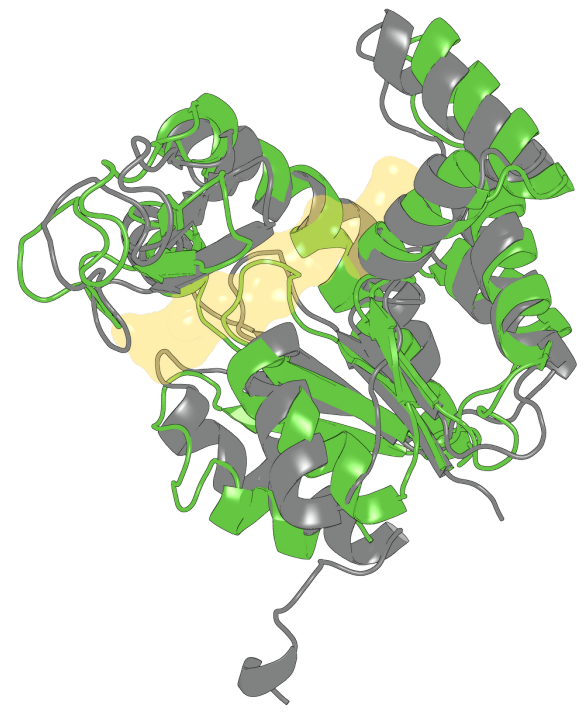 | 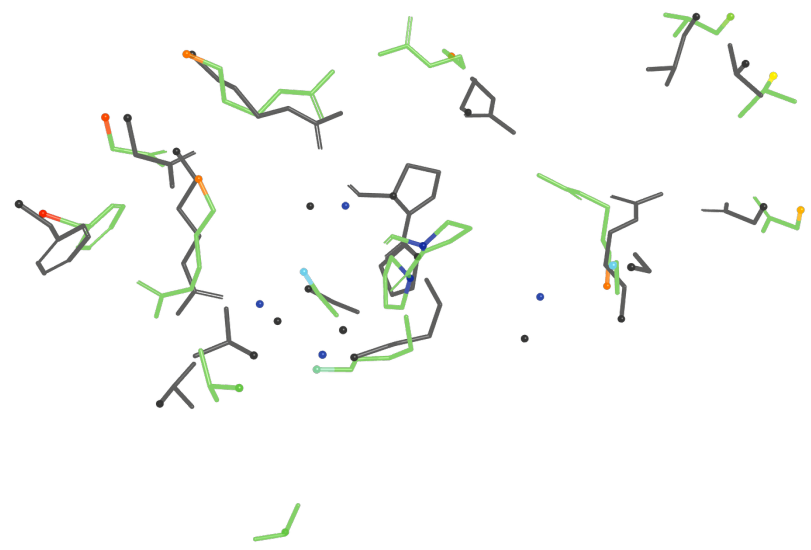 |
| | 5.1 Å | 3.0 Å |
| 15<br>ref: 2cdn (ADP) | 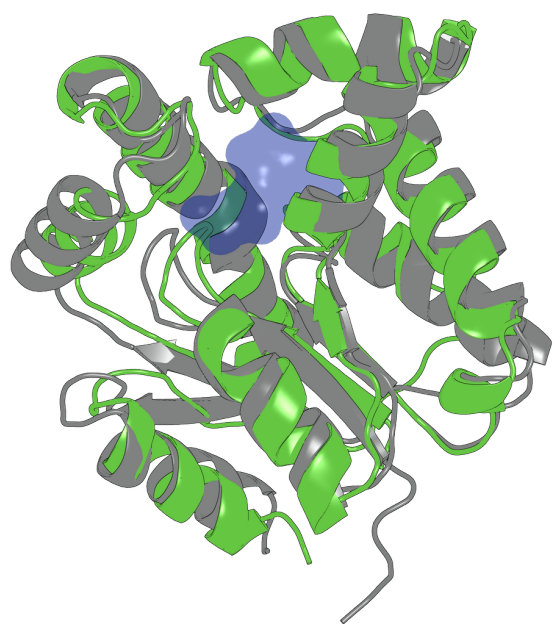 | 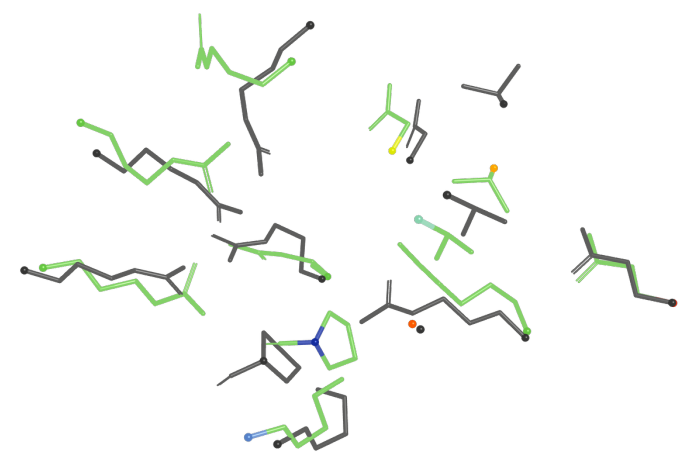 |
| | 2.6 Å | 2.2 Å |
| 15<br>ref: 2cdn (ADP) | 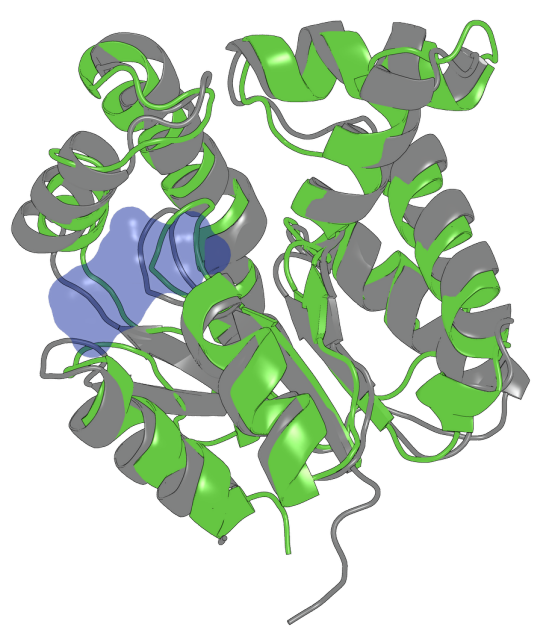 | 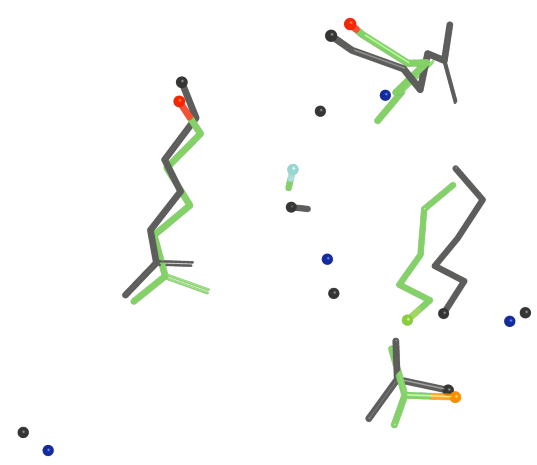 |
| | 2.7 Å | 1.2 Å |
| 16<br>ref: 5x6l (AP5) | 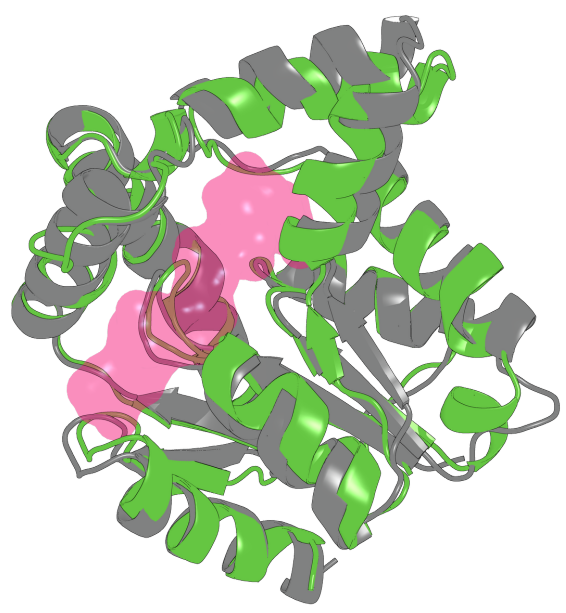 | 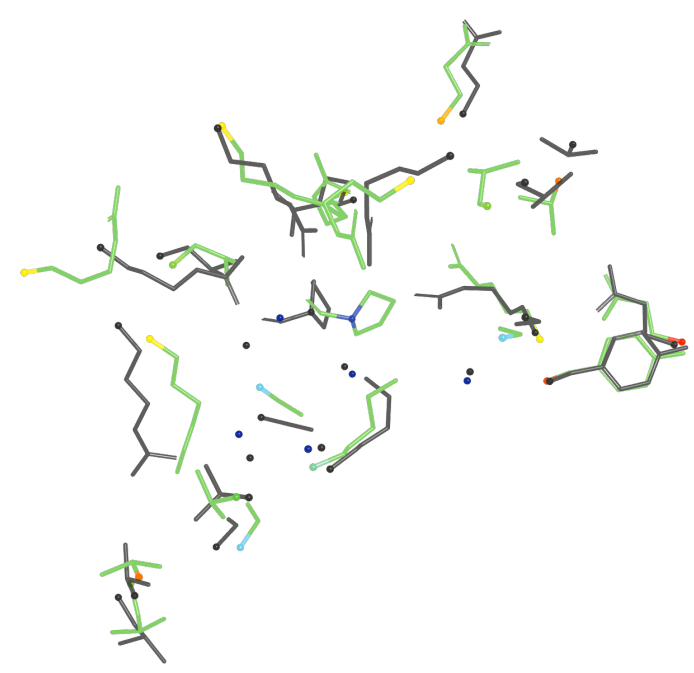 |
| | 2.0 Å | 2.1 Å |

| | | |
|---|---|---|
| 17<br>ref: 5xz2 (AP5) | 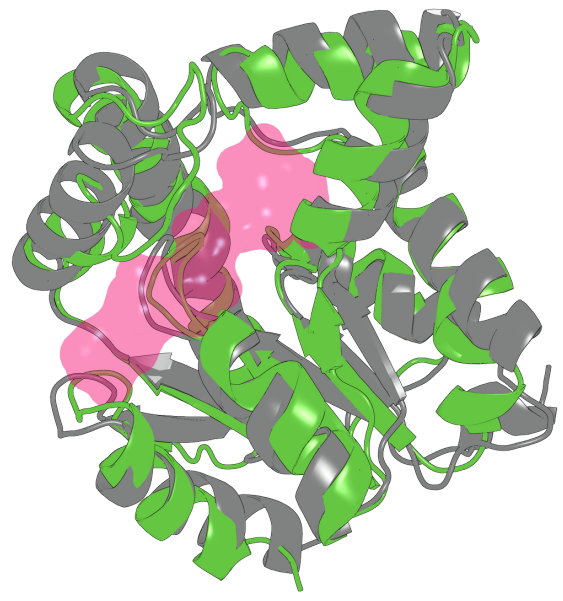 | 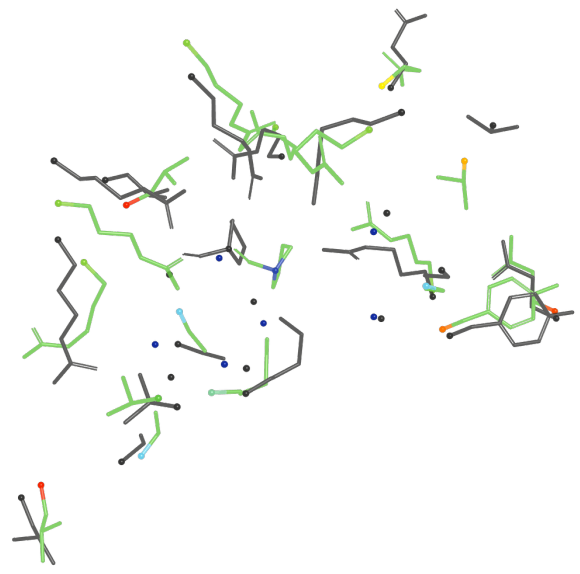 |
| | 2.2 Å | 2.1 Å |
| 18<br>ref: 5ycf (AP5) | 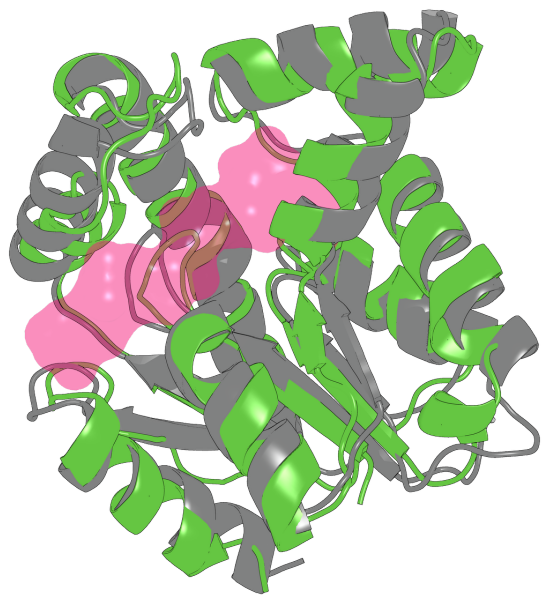 | 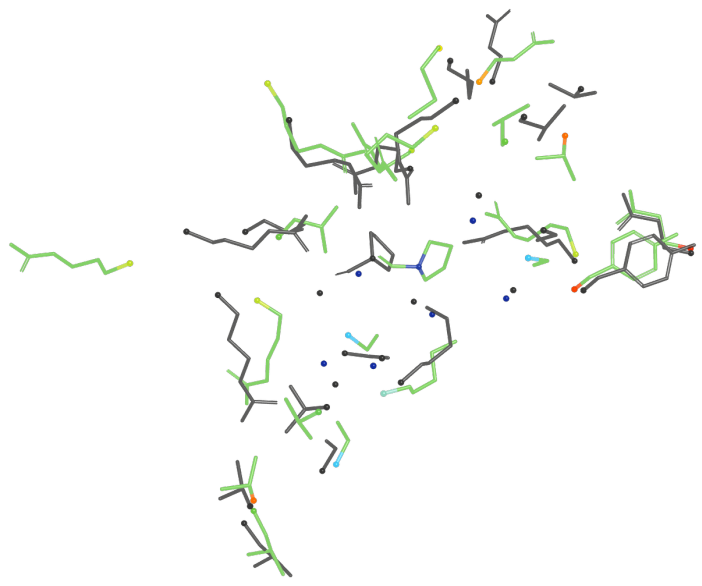 |
| | 2.2 Å | 3.1 Å |
| 19<br>ref: 5x6k (AP5) | 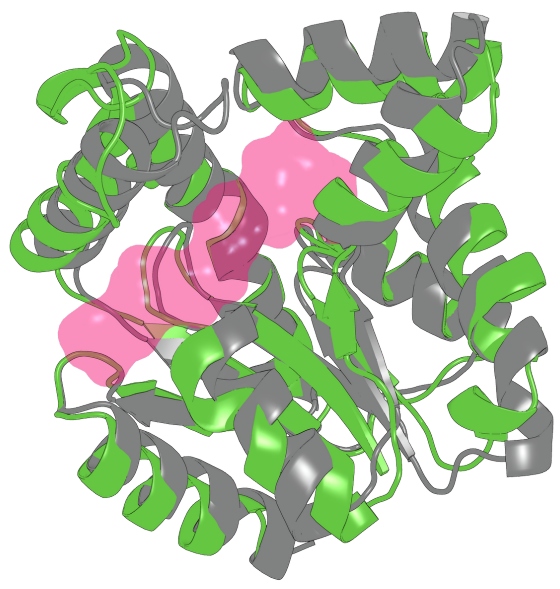 | 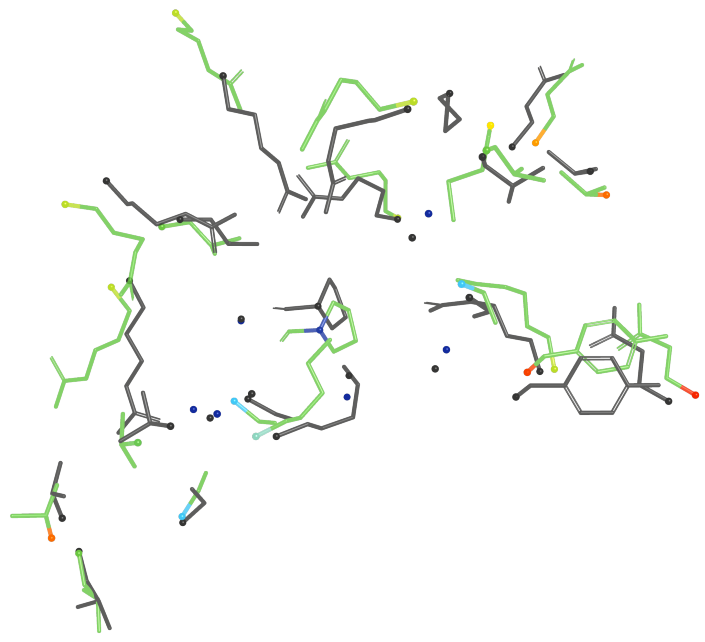 |
| | 2.5 Å | 2.7 Å |
| 20<br>ref: 5ycd (AP5) | 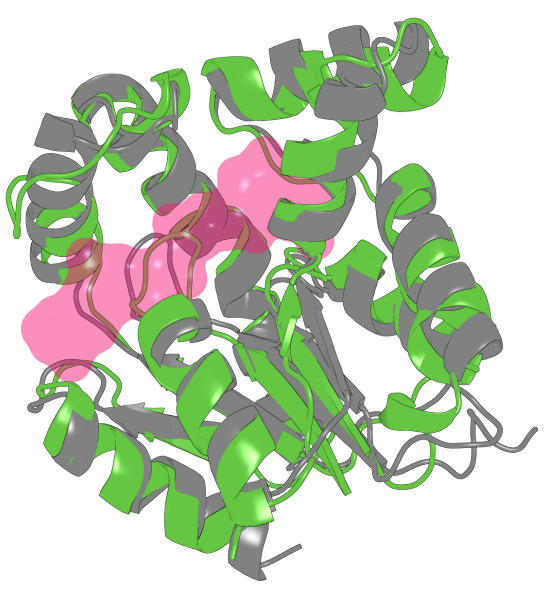 | 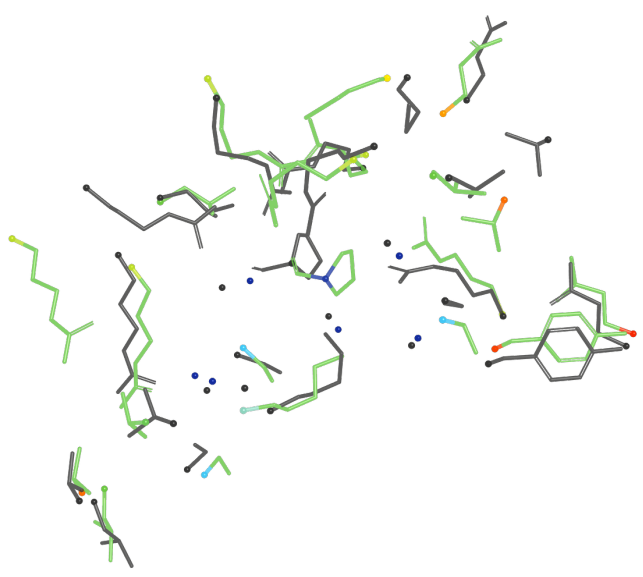 |
| | 2.2 Å | 2.4 Å |

| | | |
|---|---|---|
| 21<br>ref: 5g3y (ADP) | 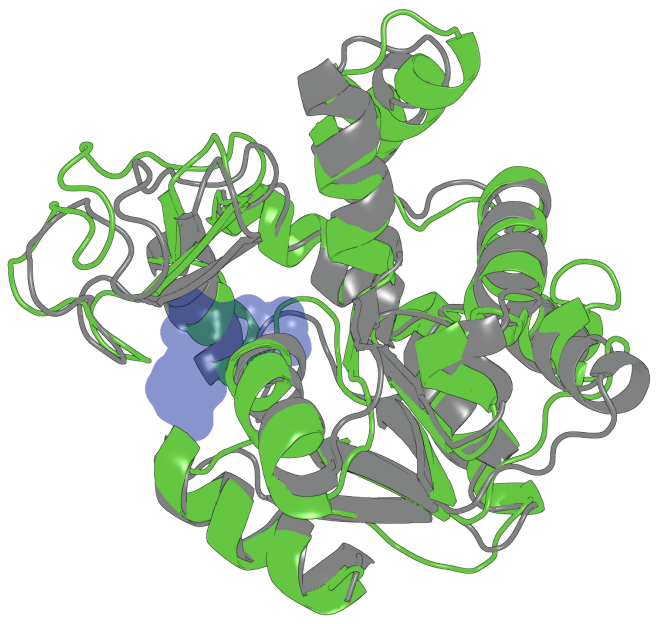 | 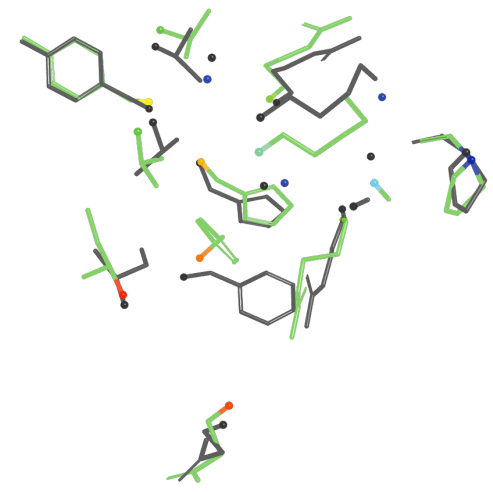 |
| | 2.5 Å | 2.0 Å |
| 21<br>ref: 5g3y (ADP) | 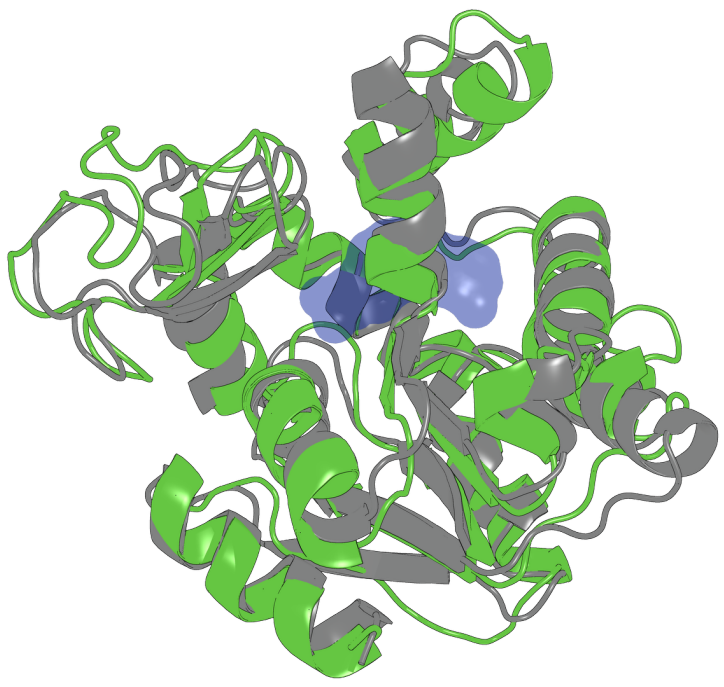 | 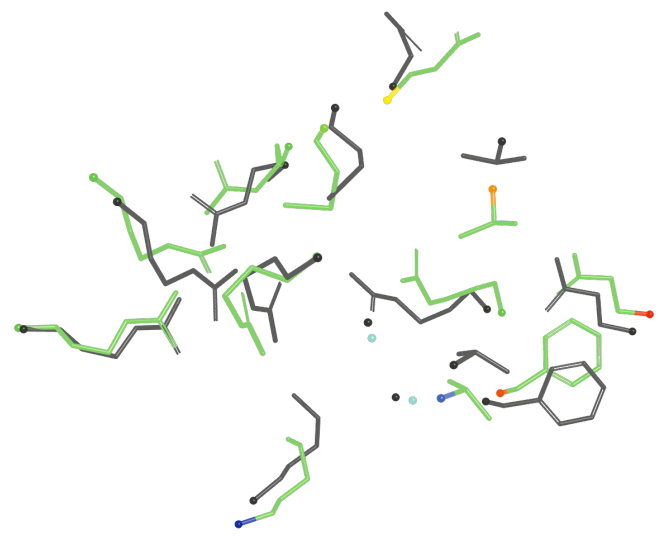 |
| | 2.5 Å | 2.0 Å |
| 22<br>ref: 5g3z (AP5) | 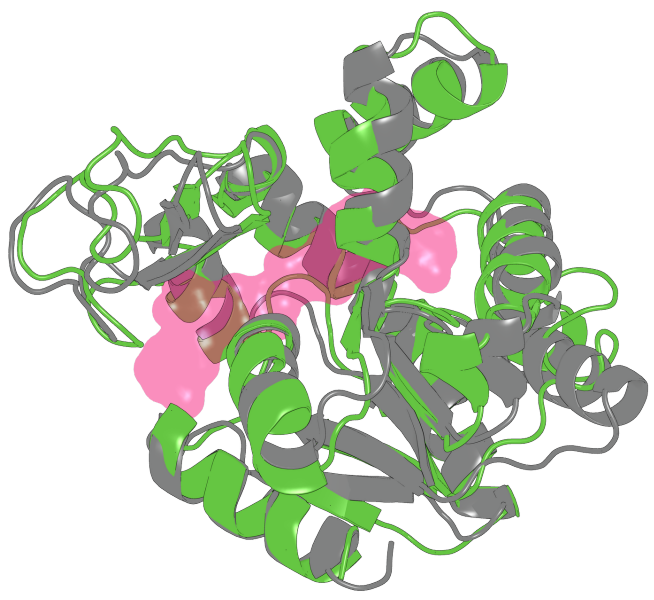 | 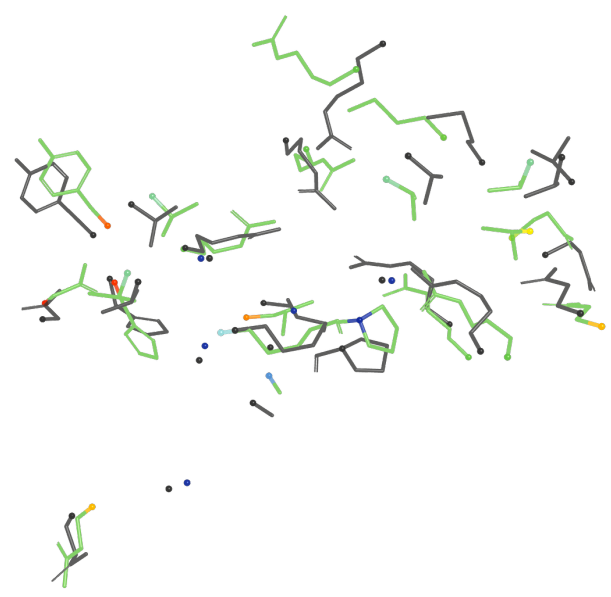 |
| | 2.4 Å | 2.1 Å |

**Figure S5. Comparison between the experimental binding modes and the top native-like conformations obtained with HADDOCK3 on the gEDES clusters.** Throughout, grey and green denote the experimental reference and homology model structures, respectively. Each system name is followed by its reference PDB ID, with the relevant ligand given in parentheses. The reported RMSD is the heavy-atoms binding mode RMSD ($RMSD_{BM}$), computed over the binding-site residues and the ligand. The experimental reference ligand is shown in grey, while the C, N, O, and P atoms of the docked ligand are colored cyan, blue, red, and orange, respectively. For clarity, only sidechain atoms are displayed. Full data for all complexes are reported in Table S6, and the corresponding values are plotted in Figure 4.

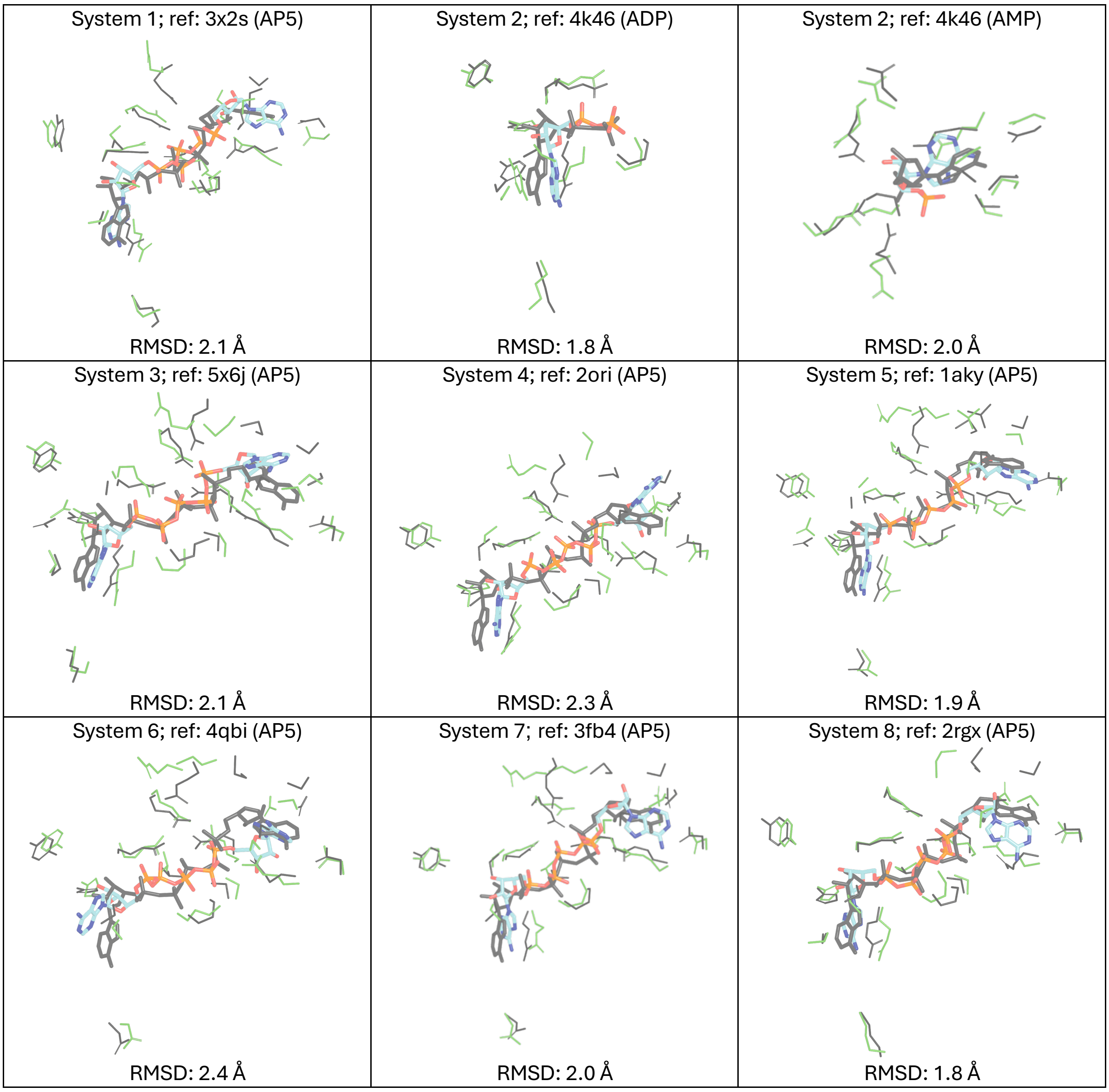

| | | |
|---|---|---|
| System 9; ref: 3tlx (ADP)<br>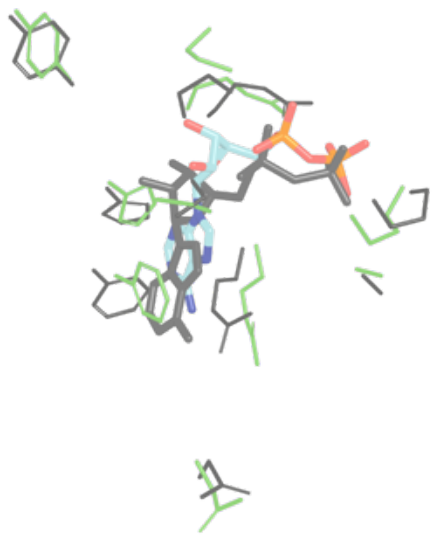<br>RMSD: 2.2 Å | System 9; ref: 3tlx (AMP)<br>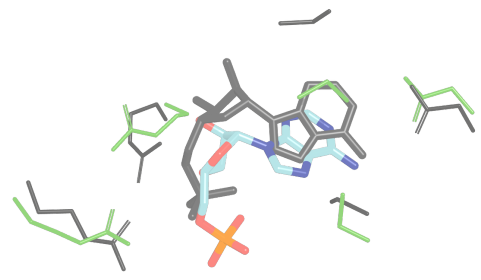<br>RMSD: 1.7 Å | System 10; ref: 1zak (AP5)<br>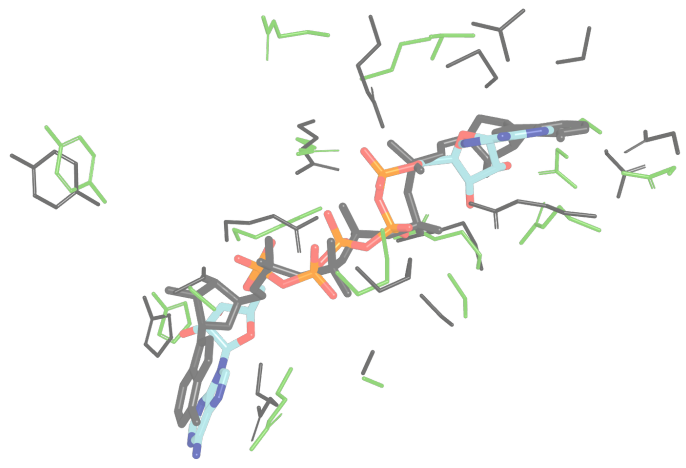<br>RMSD: 2.3 Å |
| System 11; ref: 2ak3 (AMP)<br>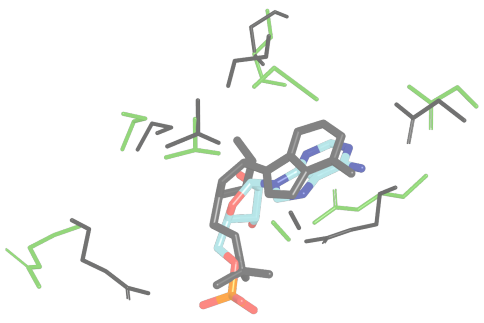<br>RMSD: 1.9 Å | System 12; ref: 6zjb (G5P)<br>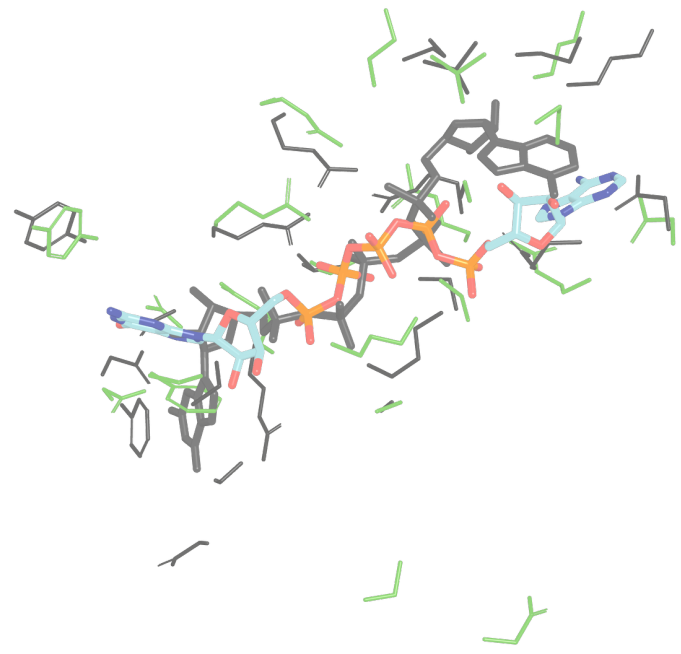<br>RMSD: 5.1 Å | System 13; ref: 4nu0 (AP5)<br>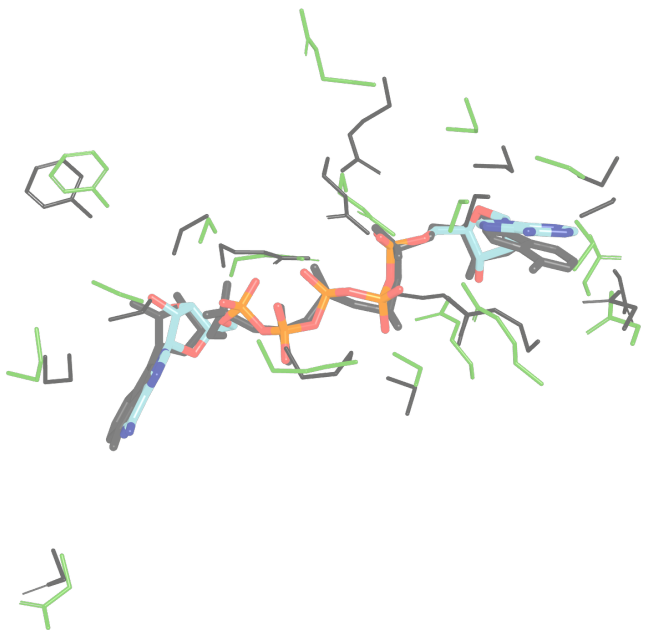<br>RMSD: 1.9 Å |
| System 14; ref: 2bbw (GP5)<br>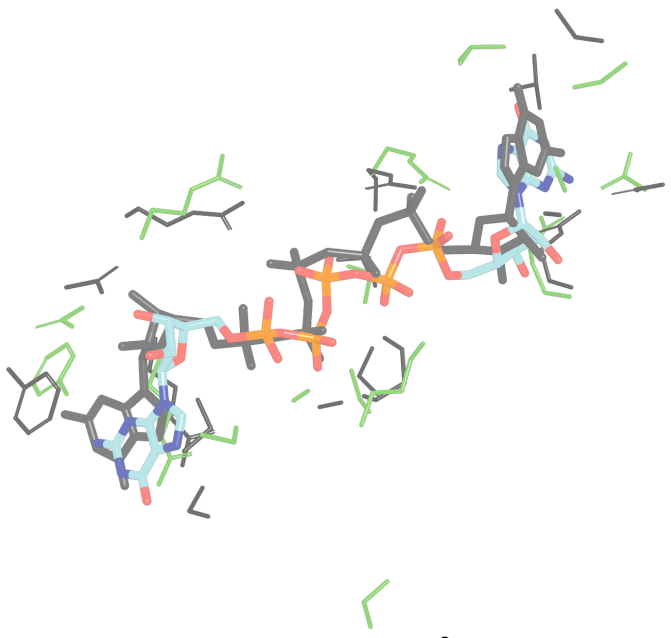<br>RMSD: 2.6 Å | System 15; ref: 2cdn (ADP)<br>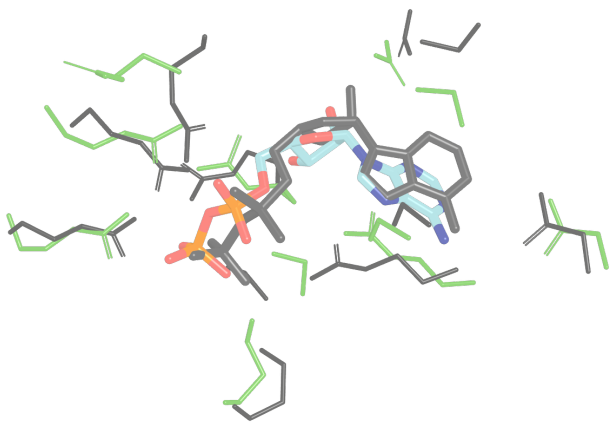<br>RMSD: 1.9 Å | System 15; ref: 2cdn (ADP)<br>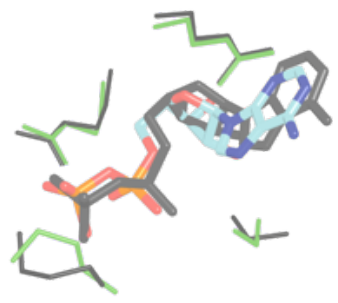<br>RMSD: 1.1 Å |
| System 16; ref: 5x6l (AP5)<br>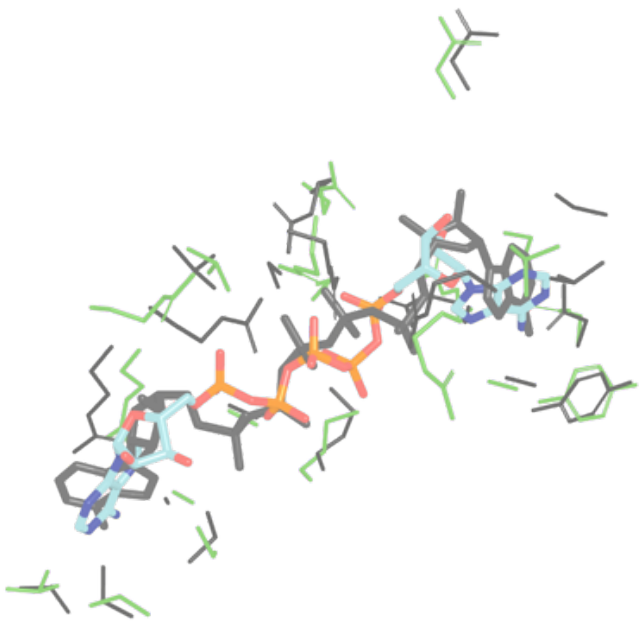<br>RMSD: 2.1 Å | System 17; ref: 5xz2 (AP5)<br>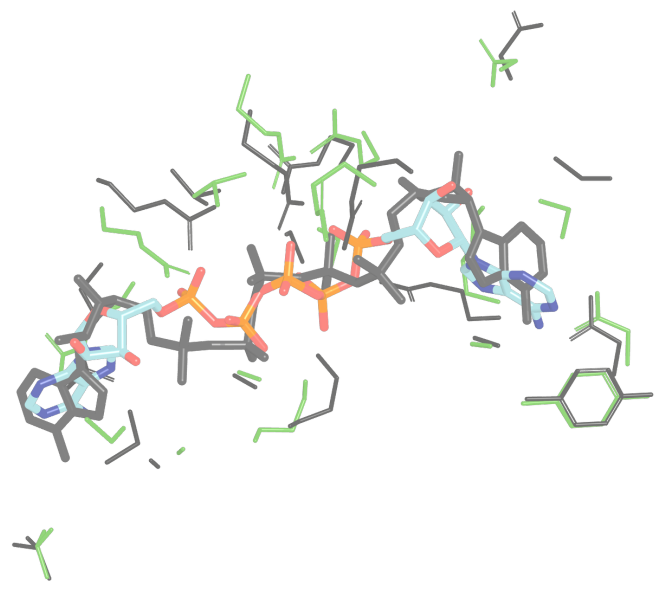<br>RMSD: 2.1 Å | System 18; ref: 5ycf (AP5)<br>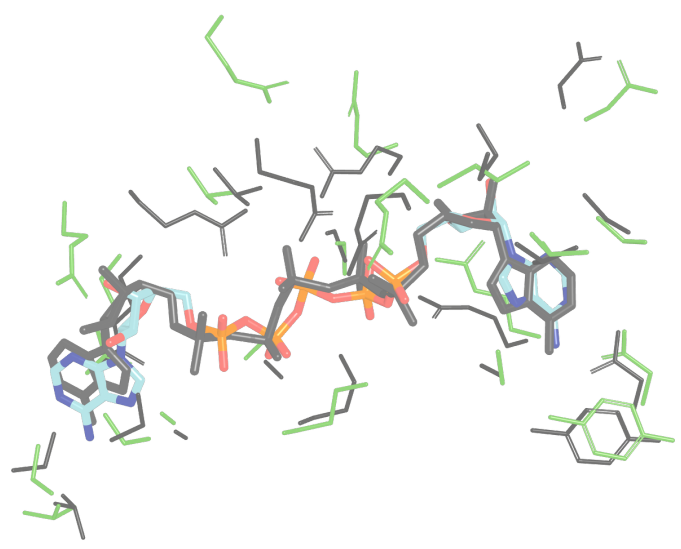<br>RMSD: 2.5 Å |

System 19; ref: 5x6k (AP5)

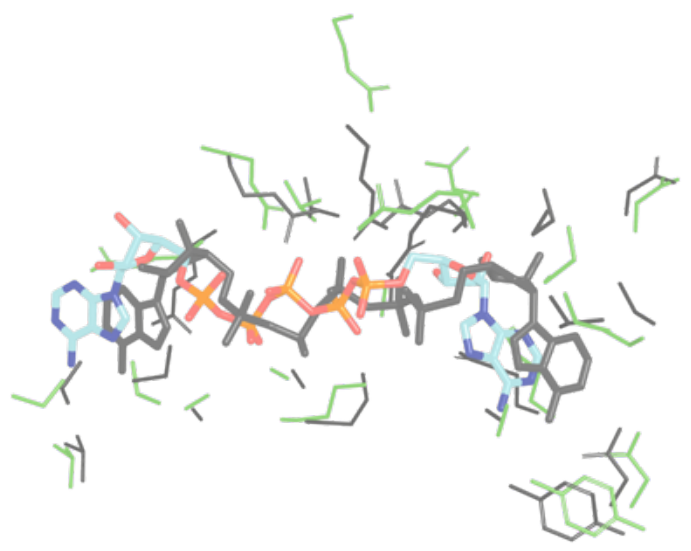

RMSD: 2.4 Å

System 20; ref: 5ycd (AP5)

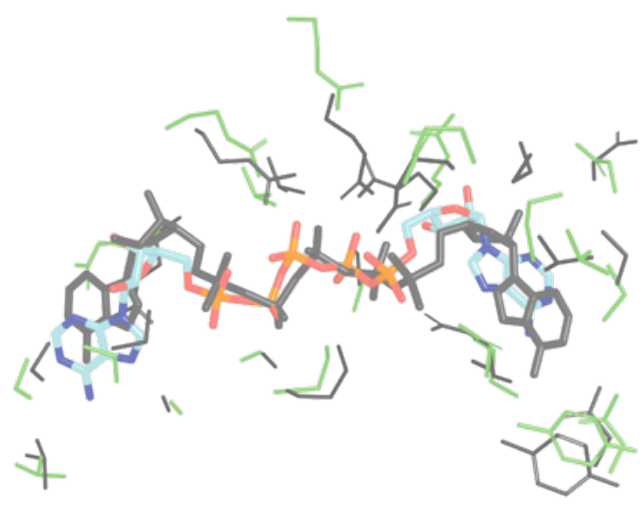

RMSD: 2.4 Å

System 21; ref: 5g3y (ADP)

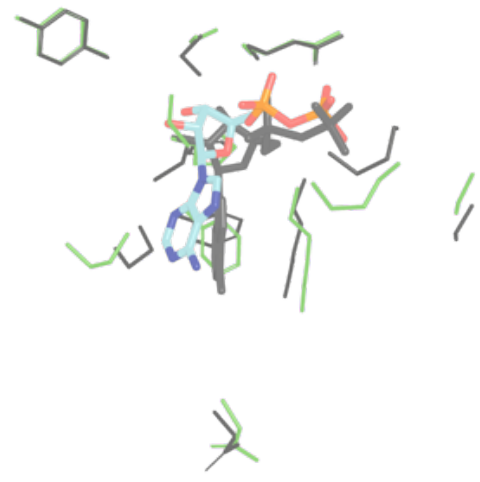

RMSD: 1.8 Å

System 21; ref: 5g3y (ADP)

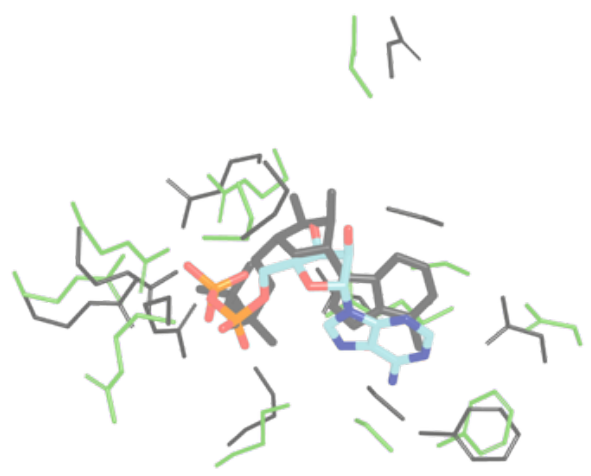

RMSD: 2.2 Å

System 22; ref: 5g3z (AP5)

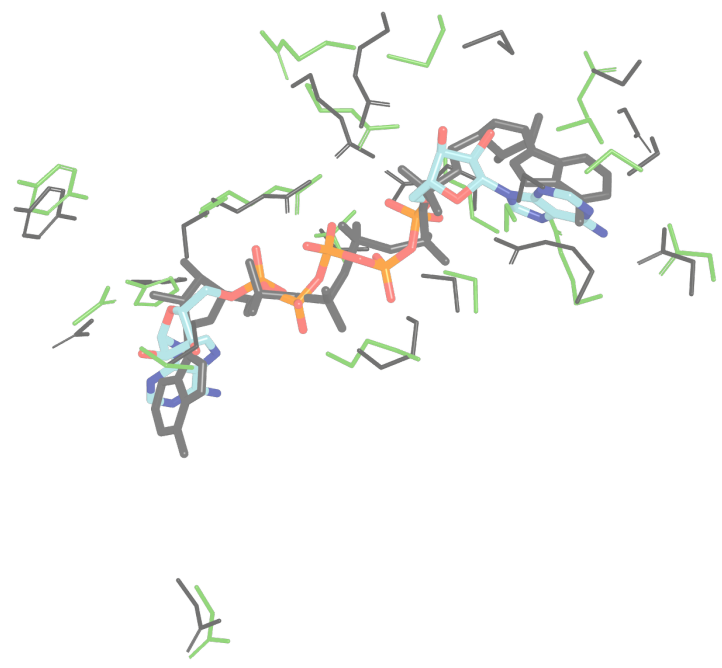

RMSD: 2.4 Å

**Figure S6.** Binding site, binding mode, and full protein accuracy of gEDES homology models (measured by their RMSD relative to the experimental references) vs. the sequence identities of the binding site (left plot) or the protein (right plot). Blue and red dots denote models below (≤ 2.5 Å) and above (> 2.5 Å) the RMSD threshold, respectively (in **A**, red points correspond to 0% of models below the cutoff). Reference-structure ligands are shown in parentheses. **(A)** Percentage of homology-models within 2.5 Å; **(B)** minimum homology-models RMSD; **(C)** RMSD of the best-scoring docking models ($RMSD_{BM}$).

**A.**

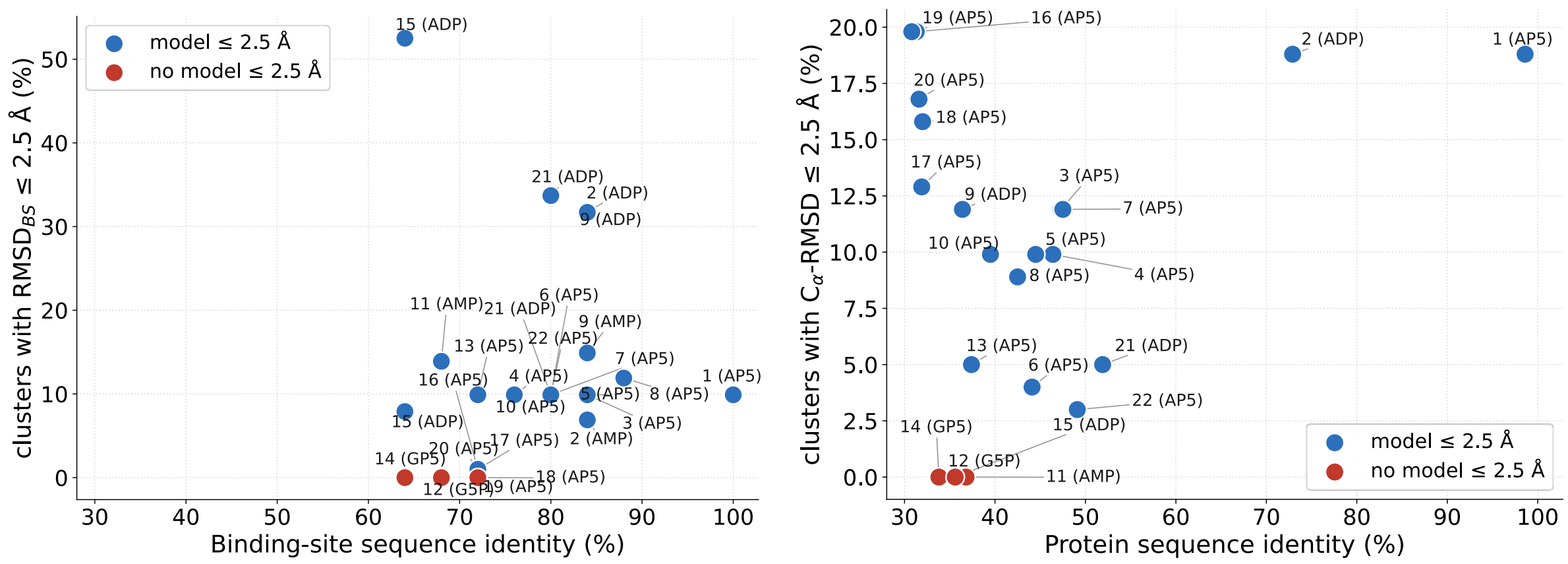


**B.**

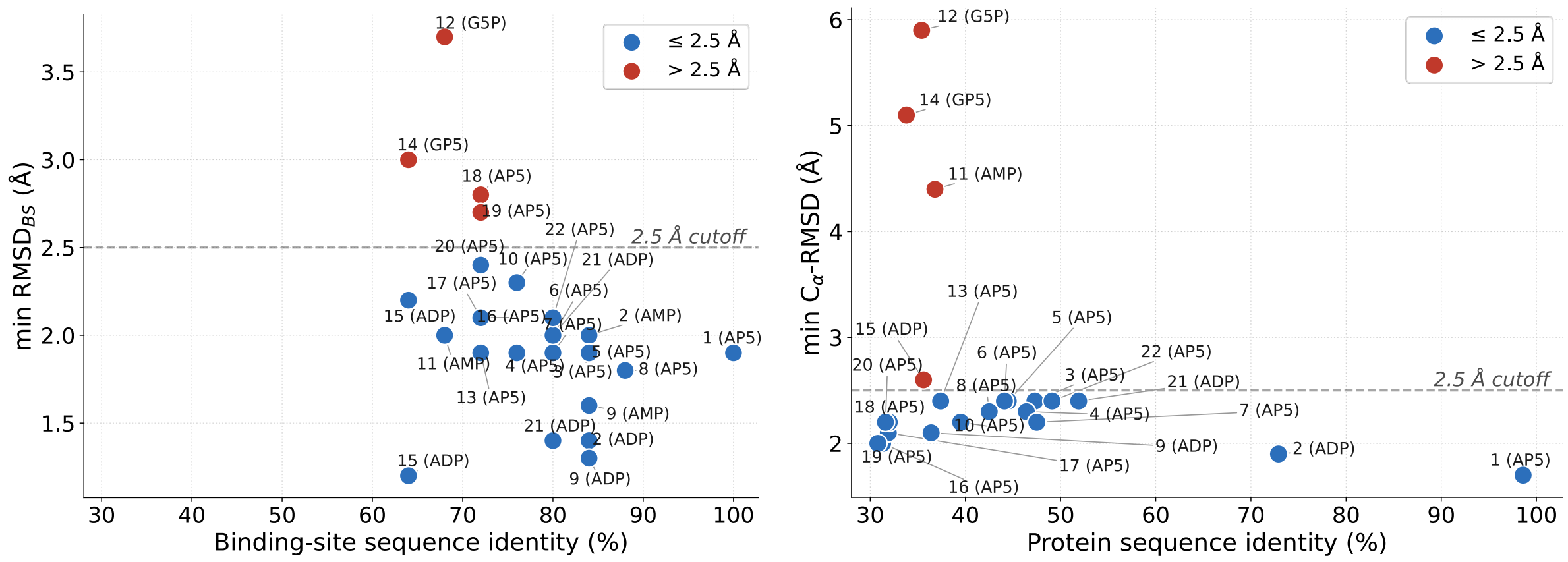


**C.**

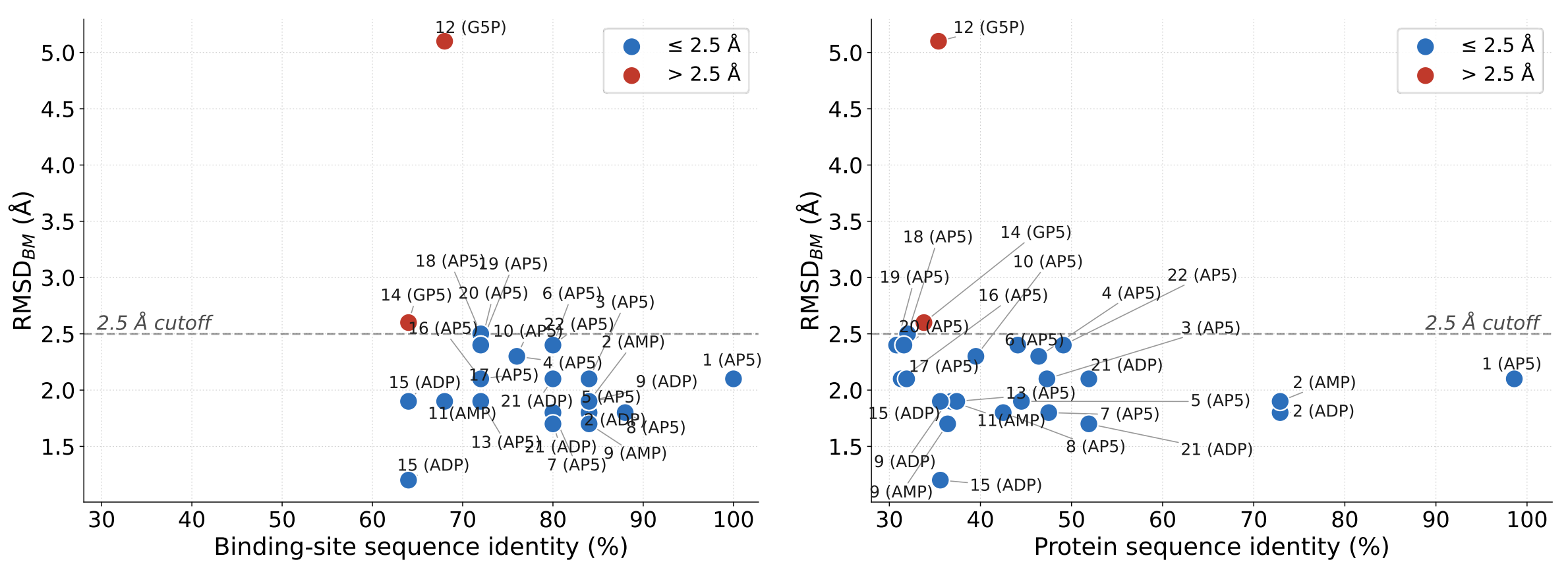

# Supplementary Tables

**Table S1. Binding-site sequence identity to the reference adenylate kinase (P69441).** BS sequence identity refers to the percentage of amino acids in the target that are identical to those in the binding site (BS) of the template, the latter being defined as the residues within 3.5 Å of the AP5 ligand in the 1AKE structure. *BS seq-ID % = (number of binding-site residues identical to ref-ADK | P69441) / 25 × 100*. Column headers give the reference residue and its P69441 position (e.g. P9, T15, R36); residues identical to the reference are shown in red, and "–" denotes an alignment gap (scored as non-identical). Alignment: Clustal Omega (v1.2.4), reformatted with MView. Binding-site positions: *"9–15, 31, 35, 36, 53, 57, 59, 64, 85, 88, 92, 123, 133, 134, 137, 156, 167, 200, 202."*

| Sequence | P9 | G10 | A11 | G12 | K13 | G14 | T15 | T31 | L35 | R36 | M53 | K57 | V59 | V64 | G85 | R88 | Q92 | R123 | Y133 | H134 | F137 | R156 | R167 | K200 | V202 | Match /25 | BS seq-ID % |
|---|---|---|---|---|---|---|---|---|---|---|---|---|---|---|---|---|---|---|---|---|---|---|---|---|---|---|---|
| **ref-ADK \| P69441** | **P** | **G** | **A** | **G** | **K** | **G** | **T** | **T** | **L** | **R** | **M** | **K** | **V** | **V** | **G** | **R** | **Q** | **R** | **Y** | **H** | **F** | **R** | **R** | **K** | **V** | **25** | **100** |
| 1 \| A0A0J9X1X4 | P | G | A | G | K | G | T | T | L | R | M | K | V | V | G | R | Q | R | Y | H | F | R | R | K | V | 25 | 100 |
| 2 \| Q6LTE1 | P | G | A | G | K | G | T | T | L | R | I | Q | V | I | G | R | Q | R | Y | H | Y | R | R | K | V | 21 | 84 |
| 3 \| P84139 | P | G | A | G | K | G | T | T | F | R | M | A | V | T | G | R | Q | R | Y | H | F | R | R | K | I | 21 | 84 |
| 4 \| P16304 | P | G | A | G | K | G | T | T | F | R | I | E | V | T | G | R | Q | R | Y | H | F | R | R | Q | I | 19 | 76 |
| 5 \| P07170 | P | G | A | G | K | G | T | T | L | R | M | G | V | M | G | R | Q | R | Y | H | F | R | R | Q | P | 21 | 84 |
| 6 \| P27142 | P | G | A | G | K | G | T | T | F | R | M | D | V | T | G | R | Q | R | Y | H | F | R | R | Q | M | 20 | 80 |
| 7 \| Q6B341 | P | G | A | G | K | G | T | T | F | R | M | N | V | T | G | R | Q | R | Y | H | F | R | R | Q | I | 20 | 80 |
| 8 \| O66490 | P | G | A | G | K | G | T | T | L | R | M | E | V | I | G | R | Q | R | Y | H | Y | R | R | K | V | 22 | 88 |
| 9 \| Q8IJV6 | P | G | S | G | K | G | T | T | L | R | I | K | V | V | G | R | Q | R | Y | H | F | R | R | Q | A | 21 | 84 |
| 10 \| P43188 | P | A | S | G | K | G | T | A | L | R | M | Q | V | V | G | R | Q | R | Y | H | Y | R | R | A | V | 19 | 76 |
| 11 \| P08760 | P | G | S | G | K | G | T | S | L | R | I | K | I | M | G | R | Q | R | Y | N | F | R | R | E | - | 17 | 68 |
| 12 \| Q9UIJ7 | P | G | S | G | K | G | T | S | L | R | I | K | I | M | G | R | Q | R | Y | N | F | R | R | E | - | 17 | 68 |
| 13 \| Q04ML5 | P | G | A | G | K | G | T | T | F | R | I | E | V | T | G | R | Q | R | F | H | F | R | R | Q | I | 18 | 72 |
| 14 \| P27144 | P | G | S | G | K | G | T | S | L | R | I | L | V | I | G | R | Q | R | Y | N | F | Q | R | E | - | 16 | 64 |
| 15 \| P9WKF5 | P | G | A | G | K | G | T | T | F | R | L | D | V | T | G | R | Q | R | - | - | - | R | R | G | M | 16 | 64 |
| 16 \| A0A2R2JFU6 | P | G | S | G | K | G | T | S | L | R | M | E | V | V | G | R | Q | R | - | - | - | R | R | L | V | 18 | 72 |
| 17 \| Q68EH2 | P | G | S | G | K | G | T | S | L | R | M | E | V | V | G | R | Q | R | - | - | - | R | R | L | V | 18 | 72 |
| 18 \| M4AA20 | P | G | S | G | K | G | T | S | L | R | M | E | V | V | G | R | Q | R | - | - | - | R | R | L | V | 18 | 72 |
| 19 \| A0A2R2JFU5 | P | G | S | G | K | G | T | S | L | R | M | E | V | V | G | R | Q | R | - | - | - | R | R | L | V | 18 | 72 |
| 20 \| A0A452CSM2 | P | G | S | G | K | G | T | S | L | R | M | E | V | V | G | R | Q | R | - | - | - | R | R | L | V | 18 | 72 |
| 21 \| 5G3Y_1 | P | G | A | G | K | G | T | T | F | R | M | E | V | T | G | R | Q | R | Y | H | F | R | R | Q | I | 20 | 80 |
| 22 \| 5G3Z_1 | P | G | A | G | K | G | T | T | F | R | M | E | V | T | G | R | Q | R | Y | H | F | R | R | Q | I | 20 | 80 |

**Table S2. Structural similarity of the homology models to the holo reference structures.** RMSD values of the models built on the gEDES cluster library ($HM_{gEDES}$), on the single experimental apo template of E. coli ADK ($HM_{4AKE(apo)}$; PDB ID 4AKE) and on the single experimental holo template ($HM_{1AKE(holo)}$; PDB ID 1AKE) are reported relative to the corresponding holo reference structures listed in Table 1. Protein RMSD was calculated on Cα atoms after Cα-based alignment, and binding-site RMSD ($RMSD_{BS}$) on non-hydrogen atoms after alignment on the binding site. Each entry gives the percentage of models with RMSD ≤ 2.5 Å, with the minimum RMSD of that set in parentheses. For $HM_{gEDES}$, results are reported separately for the 101 DOPE-selected models used as docking input and for all 1010 models generated by MODELLER[5]; for the two single-template controls all 10 models per target were included. The experimental ligand of each reference is indicated in parentheses in the first column, and the sequence identity column reports the percentage identity of each target to the reference ADK sequence (UniProt P69441). Binding-site conformations within 2.5 Å of the reference were obtained for 84.6% of the complexes with the 101 selected gEDES models, 92.3% with all gEDES models, 7.7% with the apo template and 92.3% with the holo template; the corresponding figures for the whole protein are 81.8%, 86.4%, 0% and 86.4%. Note that $HM_{1AKE(holo)}$ is not a control available in a prospective application, since it presupposes an experimental bound structure of the reference protein.

| System | Sequence Identity [%] | RMSD$_{BS}$ ≤ 2.5 Å | | | RMSD$_{prot}^{\alpha}$ ≤ 2.5 Å | | |
|---|---|---|---|---|---|---|---|
| | | selected (101 frames) / all (1010 frames) | HM$_{4AKE(apo)}$ | HM$_{1AKE(holo)}$ | selected (101 frames) / all (1010 frames) | HM$_{4AKE(apo)}$ | HM$_{1AKE(holo)}$ |
| 1<br>ref: 3x2s (AP5) | 98.6 | 9.9 (1.9) / 9.9 (1.9) | 0 (5.8) | 100 (0.8) | 18.8 (1.7) / 18.8 (1.7) | 0 (6.9) | 100 (0.6) |
| 2<br>ref: 4k46 (ADP) | 72.9 | 31.7 (1.4) / 32.8 (1.2) | 0 (3.9) | 100 (0.6) | 18.8 (1.9) / 18.8 (1.9) | 0 (7.2) | 100 (1.0) |
| 2<br>ref: 4k46 (AMP) | | 6.9 (2.0) / 7.2 (2.0) | 0 (6.0) | 100 (0.9) | | | |
| 3<br>ref: 5x6j (AP5) | 47.3 | 9.9 (1.9) / 9.9 (1.9) | 0 (5.6) | 100 (1.0) | 11.9 (2.4) / 12.2 (2.2) | 0 (6.9) | 100 (1.6) |
| 4<br>ref: 2ori (AP5) | 46.4 | 9.9 (1.9) / 9.9 (1.9) | 0 (5.7) | 100 (1.0) | 9.9 (2.3) / 10.5 (2.2) | 0 (6.6) | 100 (1.8) |
| 5<br>ref: 1aky (AP5) | 44.5 | 9.9 (1.9) / 9.9 (1.9) | 0 (5.7) | 100 (0.9) | 9.9 (2.4) / 9.9 (2.3) | 0 (7.0) | 100 (1.8) |
| 6<br>ref: 4qbi (AP5) | 44.1 | 9.9 (2.0) / 9.9 (1.9) | 0 (6.1) | 100 (0.8) | 4.0 (2.4) / 6.3 (2.3) | 0 (7.3) | 100 (1.8) |
| 7<br>ref: 3fb4 (AP5) | 47.5 | 9.9 (1.9) / 9.9 (1.9) | 0 (5.8) | 100 (1.1) | 11.9 (2.2) / 12.6 (2.2) | 0 (6.7) | 100 (1.8) |
| 8<br>ref: 2rgx (AP5) | 42.5 | 11.9 (1.8) / 11.8 (1.7) | 0 (5.5) | 100 (1.1) | 8.9 (2.3) / 8.9 (2.2) | 0 (6.4) | 100 (2.0) |
| 9<br>ref: 3tlx (ADP) | 36.4 | 31.7 (1.3) / 30.4 (1.3) | 0 (3.7) | 100 (0.9) | 11.9 (2.1) / 10.3 (2.1) | 0 (7.7) | 100 (2.1) |
| 9<br>ref: 3tlx (AMP) | | 14.9 (1.6) / 15.2 (1.5) | 0 (4.8) | 100 (0.9) | | | |
| 10<br>ref: 1zak (AP5) | 39.5 | 9.9 (2.3) / 9.0 (2.2) | 0 (6.0) | 100 (1.1) | 9.9 (2.2) / 9.7 (2.2) | 0 (6.6) | 100 (1.8) |
| 11<br>ref: 2ak3 (AMP) | 36.8 | 13.9 (2.0) / 13.2 (1.9) | 80 (2.3) | 100 (0.8) | 0 (4.4) / 0 (4.3) | 0 (5.0) | 0 (7.7) |
| 12<br>ref: 6zjb (G5P) | 35.4 | 0 (3.7) / 0 (3.7) | 0 (6.2) | 0 (3.4) | 0 (5.9) / 0 (5.7) | 0 (8.2) | 0 (6.0) |
| 13<br>ref: 4nu0 (AP5) | 37.4 | 9.9 (1.9) / 9.9 (1.9) | 0 (5.9) | 100 (1.0) | 5.0 (2.4) / 5.3 (2.4) | 0 (6.4) | 100 (1.9) |
| 14<br>ref: 2bbw (GP5) | 33.8 | 0 (3.0) / 0 (3.0) | 0 (4.0) | 0 (3.0) | 0 (5.1) / 0 (5.1) | 0 (6.2) | 0 (5.8) |
| 15<br>ref: 2cdn (ADP-1) | 35.6 | 7.9 (2.2) / 6.2 (2.2) | 0 (7.1) | 90 (1.5) | 0 (2.6) / 0.2 (2.5) | 0 (5.7) | 100 (2.2) |
| 15<br>ref: 2cdn (ADP-2) | | 52.5 (1.2) / 53.8 (1.1) | 30 (2.5) | 100 (0.7) | | | |
| 16<br>ref: 5x6l (AP5) | 31.3 | 1.0 (2.1) / 0.4 (2.1) | 0 (4.9) | 70 (1.3) | 19.8 (2.0) / 19.3 (2.0) | 0 (5.1) | 100 (2.0) |
| 17<br>ref: 5xz2 (AP5) | 31.9 | 1.0 (2.1) / 0.4 (2.1) | 0 (4.9) | 60 (1.3) | 12.9 (2.1) / 13.0 (2.1) | 0 (5.2) | 100 (2.0) |
| 18<br>ref: 5ycf (AP5) | 32.0 | 0 (2.8) / 0.5 (2.0) | 0 (6.0) | 80 (1.2) | 15.8 (2.2) / 15.7 (2.1) | 0 (5.4) | 100 (2.0) |
| 19<br>ref: 5x6k (AP5) | 30.8 | 0 (2.7) / 0.2 (2.4) | 0 (5.6) | 70 (1.4) | 19.8 (2.0) / 19.4 (2.0) | 0 (5.4) | 100 (2.0) |
| 20<br>ref: 5ycd (AP5) | 31.6 | 1.0 (2.4) / 0.4 (2.0) | 0 (5.5) | 50 (1.3) | 16.8 (2.2) / 15.4 (2.1) | 0 (5.4) | 100 (2.0) |
| 21<br>ref: 5g3y (ADP-1) | 51.9 | 33.7 (1.4) / 35.1 (1.2) | 0 (3.5) | 100 (0.7) | 5.0 (2.4) / 4.0 (2.4) | 0 (7.4) | 100 (1.7) |
| 21<br>ref: 5g3y (ADP-2) | | 9.9 (2.0) / 9.7 (1.9) | 0 (6.2) | 100 (0.8) | | | |
| 22<br>ref: 5g3z (AP5) | 49.1 | 9.9 (2.1) / 9.9 (2.0) | 0 (5.7) | 100 (1.2) | 3.0 (2.4) / 3.4 (2.4) | 0 (6.6) | 100 (1.9) |
| Overall %<br>native-like conformations | | 84.6 / 92.3 | 7.7 | 92.3 | 81.8 / 86.4 | 0 | 86.4 |

**Table S3. Projection of the reference structures, of the gEDES representatives, of the homologue holo structures and of seven experimental structures representing putative intermediate conformations onto the two collective coordinates describing the open-to-close ADK transition.** The distances $d_1$ and $d_2$ are in Å, respectively defined between the centers-of-mass of the LID (residues 118-160) and CORE (1-29, 68-117 and 161-214) and NMP (30-67) and CORE domains (residue numbering according to *E. coli* sequence). The angles $\theta_1$ and $\theta_2$ are in degrees, defined as in ref.[6]: namely, $\theta_1$ is the angle formed by the centers of mass of the backbone of residues 123-155 (LID), 161-165 (hinge), and 1-8, 79-85, 104-110, and 190-198 (CORE); $\theta_2$ is formed by the centers of mass of the backbone of residues of 50-59 (NMP), CORE, and hinge. Both sets of definitions were mapped onto each homologue structure through the alignment used to place it in *E. coli* numbering, with the residue-numbering offset determined empirically. Systems 15 to 20 lack between 63 and 72% of the LID residues, so their LID coordinates were computed over the surviving fraction of the domain. For systems 8, 10 and 13 the LID-involving distances and angles are computed respectively over 84, 91, 88% and 79, 88 and 85% of the LID residues, the remainder having no counterpart in their natively shorter LID insertion.

| Structure or set | $d_1$ [Å] | $d_2$ [Å] | $\theta_1$ [°] | $\theta_2$ [°] | Note |
|---|---|---|---|---|---|
| **4AKE (open, apo)** | 29.6 | 21.8 | 94.8 | 61.5 | Experimental endpoint, reference |
| **1AKE (closed, holo)** | 20.5 | 18.3 | 62.8 | 27.0 | Experimental endpoint, reference |
| **1ZIN** | 22.7 | 18.4 | 70.3 | 28.7 | Ap5A, partly open LID, closed NMP |
| **1ZIO** | 22.9 | 18.4 | 71.1 | 28.6 | Mg-Ap5A, partly open LID, closed NMP |
| **1ZIP** | 22.8 | 18.4 | 70.5 | 28.6 | Mn-Ap5A, partly open LID, closed NMP |
| **1AK2** | 25.2 | 20.7 | 65.5 | 59.9 | Semi-open-semi-closed, closed LID, open NMP |
| **2AK2** | 25.0 | 20.6 | 65.6 | 59.7 | Semi-open-semi-closed, closed LID, open NMP |
| **1DVR** | 22.2 | 21.0 | 64.1 | 59.8 | Semi-open-semi-closed, closed LID, open NMP |
| **2C9Y** | 26.5 | 20.7 | 70.2 | 60.1 | Semi-open-semi-closed, closed LID, open NMP |
| ***E. coli* ADK gEDES representatives (n = 101)** | 17.5-32.0 | 17.5-22.1 | 55.9-120.9 | 28.0-67.1 | From Basciu *et al.*, *J. Chem. Inf. Model.* (2025) 65:2052 |
| **3TLX (#9)** | 19.11 | 17.89 | 62.9 | 28.0 | |
| **5G3Y (#21)** | 20.00 | 18.30 | 59.9 | 27.4 | |
| **6ZJB (#12)** | 20.05 | 17.43 | 63.9 | 29.1 | unrecovered complex |
| **4K46 (#2)** | 20.14 | 18.72 | 61.1 | 27.2 | |
| **1AKY (#5)** | 20.38 | 18.43 | 64.5 | 26.1 | |
| **3X2S (#1)** | 20.47 | 18.19 | 63.4 | 27.0 | |
| **2RGX (#8)** | 20.69 | 18.45 | 67.8 | 28.1 | Short-form, see caption |
| **5X6J (#3)** | 20.71 | 18.35 | 62.9 | 28.3 | |
| **4QBI (#6)** | 20.73 | 18.57 | 60.5 | 27.9 | |
| **3FB4 (#7)** | 20.81 | 18.31 | 64.1 | 28.4 | |
| **5G3Z (#22)** | 20.87 | 18.21 | 61.7 | 27.6 | |
| **2ORI (#4)** | 20.89 | 18.24 | 63.3 | 27.8 | |
| **4NU0 (#13)** | 21.46 | 18.30 | 64.8 | 27.8 | Short-form, see caption |
| **1ZAK (#10)** | 21.62 | 18.80 | 67.1 | 31.1 | Short-form, see caption |
| **2BBW (#14)** | 24.05 | 21.70 | 77.4 | 44.1 | unrecovered complex, between γ and α basins |
| **2AK3 (#11)** | 30.38 | 19.60 | 98.2 | 38.7 | AMP-only complex, LID open |
| **2CDN, 5X6L, 5XZ2, 5YCF, 5X6K, 5YCD (#15-20)** | n.d. | 15.53-18.69 | n.d. | 27.2-28.7 | Short-forms, see caption |

**Table S4. Closest member of the transferred pool for each long-form homologue structure.** The nearest representative is the member of the 101 that minimizes the $C_\alpha$-RMSD over the LID domain, with the LID defined as in Table S3. The comparison is made after placing all structures in a common reference frame built from the experimental structures alone, and not by superposing each pair individually.

| System | Nearest representative (LID) | min $C_\alpha$-RMSD, LID [Å] |
|---|---|---|
| 1 | gEDES_035 | 1.59 |
| 2 | gEDES_064 | 1.70 |
| 3 | gEDES_055 | 1.69 |
| 4 | gEDES_063 | 1.56 |
| 5 | gEDES_055 | 1.38 |
| 6 | gEDES_063 | 1.77 |
| 7 | gEDES_055 | 1.54 |
| 8 | gEDES_050 | 2.42 |
| 9 | gEDES_035 | 1.17 |
| 10 | gEDES_063 | 2.62 |
| 11 | gEDES_086 | 4.41 |
| 12 | gEDES_044 | 1.61 |
| ` | gEDES_050 | 2.72 |
| 14 | gEDES_045 | 1.88 |
| 15 | gEDES_056 | 1.71 |
| 16 | gEDES_066 | 5.19 |
| 17 | gEDES_066 | 5.44 |
| 18 | gEDES_066 | 5.03 |
| 19 | gEDES_066 | 5.14 |
| 20 | gEDES_066 | 5.13 |
| 21 | gEDES_035 | 1.84 |
| 22 | gEDES_063 | 1.86 |

**Table S5. HADDOCK3 ensemble cross-docking workflow.** Modules are executed sequentially in the order shown. Values not stated explicitly in the configuration file (mdsteps_cool2, nemsteps, and the rigid-body desolvation/restraint weights) are HADDOCK3[7] defaults resolved at run time and are flagged as such. Sampling was scaled so that each of the 101 conformers was sampled ~250 times (25250 models total).

| Module | Purpose | Key settings |
|---|---|---|
| `topoaa` | Topology generation for every ensemble member and the ligand | `ligand_top_fname = "ligand.top";` `ligand_param_fname = "ligand.param";` `delenph = false; autohis = true;` `set_bfactor = false` |
| `rigidbody` | Rigid-body docking; 250 models per ensemble conformer | `sampling = 25250; tolerance = 20; randorien = true;` `rigidtrans = true; ntrials = 5;` `mol_fix_origin_2 = false` `ambig_fname = "ambig_active.tbl";` `inter_rigid = 0.001` *(default 1.0)*`;` `w_vdw = 0.0` *(default 0.01)*`; w_elec = 1.0;` `w_desolv = 1.0; w_air = 0.01; w_bsa = -0.01;` `cmrest = true; cmtight = true;` `mol_fix_origin_1 = true; randremoval = false` |
| `seletop` | Score-based selection (global, not per conformer) | `select = 5050` |
| `flexref` | Semi-flexible refinement; binding-site side chains only (padded by ±1 and merged into segments per target), ligand rigid | `tolerance = 50; ambig_fname = "ambig_active.tbl";` `nseg2 = 0; randremoval = false;` `mdsteps_rigid = 0; mdsteps_cool1 = 0;` `mdsteps_cool3 = 0;` `mdsteps_cool2 = 1000;` `sinter_cool2: 0.001 -> 1.0;` `semi-flexible segments defined per target` *(seg_sta_1_i / seg_end_1_i)* |
| `emref` | Energy-minimisation refinement (CNS conjugate gradient); final refinement stage used in this work | `tolerance = 5; ambig_fname = "ambig_active.tbl";` `nemsteps = 200` |
| `caprieval` | CAPRI evaluation of the emref-refined models against the reference (i-RMSD, l-RMSD, Fnat, DockQ) | `reference_fname = "reference.pdb";` `irmsd_cutoff = 3.5` |

**Table S6. Native-like poses identified by HADDOCK.** For each complex the table reports the representative pose of the cluster containing the best native-like solution, given binding mode, ligand, binding site, and whole protein as $RMSD_{BM/lig/BS/prot}$ with the corresponding $F_{nat}$ on the following line; all five quantities in a given entry therefore refer to one and the same pose. The value in parentheses is the rank of that cluster in the HADDOCK score ranking, clusters being ranked by the mean of their three best scores. Four receptor conditions are compared, all of them using the same docking protocol, restraints and post-processing: the gEDES-derived ensembles ($HM_{gEDES}$, 101 models per target), the models built on the single experimental apo template ($HM_{4AKE(apo)}$, 10 models per target), the models built on the single experimental holo template ($HM_{1AKE(holo)}$, 10 models per target), and the experimental holo receptors themselves with the ligand removed ($HOLO_{expt}$, redocking). $F_{nat}$ was computed over the residues within 5 Å of the ligand in the reference structure. Protein RMSD used Cα atoms for both alignment and calculation, all other RMSD metrics non-hydrogen atoms. The experimental ligand of each reference is indicated in parentheses in the first column. Native-like binding modes ($RMSD_{BM} \leq 2.5$ Å) were obtained for 92.3% of the complexes with $HM_{gEDES}$, 7.7% with $HM_{4AKE(apo)}$, 92.3% with $HM_{1AKE(holo)}$ and 96.2% with $HOLO_{expt}$; the median $RMSD_{BM}$ is 2.1, 5.1, 1.1 and 0.6 Å respectively. The redocking control defines the practical ceiling of the docking protocol on this ligand set and the noise floor of the RMSD metrics.

| System | HADDOCK3 | | | |
|---|---|---|---|---|
| | $\mathbf{HM_{gEDES}}$ | $\mathbf{HM_{4AKE(apo)}}$ | $\mathbf{HM_{1AKE(holo)}}$ | $\mathbf{HOLO_{expt}}$ |
| 1<br>ref: 3x2s (AP5) | 2.1/2.0/2.0/2.1 (5)<br>0.76 | 5.2/2.1/5.8/7.0 (2)<br>0.40 | 1.0/0.6/1.0/0.6 (1)<br>0.98 | 1.7/3.3/0.8/0.3 (2)<br>0.77 |
| 2<br>ref: 4k46 (ADP) | 1.8/1.3/2.0/2.1 (1)<br>0.89 | 3.6/2.3/3.9/7.5 (1)<br>0.58 | 0.7/0.6/0.7/1.0 (1)<br>0.89 | 0.6/0.7/0.5/0.2 (1)<br>0.96 |
| 2<br>ref: 4k46 (AMP) | 2.0/2.4/1.8/2.1 (1)<br>0.87 | 5.7/5.0/5.8/7.6 (5)<br>0.52 | 1.0/0.9/0.8/1.0 (1)<br>0.87 | 0.5/0.6/0.5/0.2 (1)<br>0.91 |
| 3<br>ref: 5x6j (AP5) | 2.1/2.3/2.0/2.4 (1)<br>0.83 | 5.1/2.1/5.7/7.0 (5)<br>0.49 | 1.0/0.7/1.0/1.6 (1)<br>0.92 | 1.4/2.7/0.7/0.2 (1)<br>0.85 |
| 4<br>ref: 2ori (AP5) | 2.3/3.4/2.0/2.4 (3)<br>0.72 | 5.1/3.8/5.5/6.6 (4)<br>0.47 | 1.1/0.8/1.1/1.8 (1)<br>0.94 | 0.5/0.6/0.5/0.2 (1)<br>0.94 |
| 5<br>ref: 1aky (AP5) | 1.9/1.8/1.9/2.3 (10)<br>0.83 | 5.3/2.1/5.9/7.1 (6)<br>0.50 | 1.0/0.7/0.9/1.8 (1)<br>0.95 | 2.4/5.2/0.5/0.2 (1)<br>0.79 |
| 6<br>ref: 4qbi (AP5) | 2.4/3.4/2.0/2.4 (4)<br>0.74 | 5.3/1.9/6.0/7.5 (7)<br>0.46 | 1.0/0.8/1.0/1.8 (1)<br>0.89 | 0.5/0.6/0.4/0.2 (1)<br>0.91 |
| 7<br>ref: 3fb4 (AP5) | 2.0/2.5/1.9/2.2 (5)<br>0.67 | 5.2/3.3/5.7/6.7 (7)<br>0.46 | 1.1/0.7/1.1/2.0 (1)<br>0.88 | 2.4/5.1/0.8/0.4 (1)<br>0.69 |
| 8<br>ref: 2rgx (AP5) | 1.8/2.3/1.6/2.3 (1)<br>0.72 | 4.8/1.8/5.4/6.5 (9)<br>0.45 | 1.1/0.8/1.1/2.2 (1)<br>0.92 | 0.5/0.7/0.4/0.2 (1)<br>0.98 |
| 9<br>ref: 3tlx (ADP) | 2.2/2.4/2.0/2.4 (4)<br>0.62 | 3.4/2.0/3.6/7.8 (1)<br>0.54 | 1.1/0.6/1.0/2.1 (1)<br>0.81 | 0.5/0.7/0.4/0.1 (3)<br>1.0 |
| 9<br>ref: 3tlx (AMP) | 1.7/2.0/1.6/2.4 (11)<br>0.80 | 5.2/5.8/5.0/7.8 (4)<br>0.25 | 0.8/0.7/0.8/2.1 (1)<br>0.90 | 1.1/1.7/0.8/0.1 (1)<br>0.90 |
| 10<br>ref: 1zak (AP5) | 2.3/2.0/2.3/2.3 (1)<br>0.81 | 5.5/3.3/6.0/6.7 (2)<br>0.50 | 1.1/0.6/1.2/1.8 (1)<br>0.93 | 0.6/0.6/0.6/0.2 (1)<br>0.93 |
| 11<br>ref: 2ak3 (AMP) | 1.9/1.3/2.0/6.7 (4)<br>0.88 | 2.5/3.1/2.2/5.1 (9)<br>0.75 | 0.9/0.7/0.8/7.7 (1)<br>1.0 | 0.5/0.7/0.5/0.2 (1)<br>0.88 |
| 12<br>ref: 6zjb (G5P) | 5.1/5.5/5.0/6.1 (2)<br>0.75 | 6.2/2.4/6.8/8.2 (4)<br>0.46 | 4.9/6.1/4.5/6.1 (4)<br>0.66 | 2.9/0.6/3.1/0.2 (1)<br>0.96 |
| 13<br>ref: 4nu0 (AP5) | 1.9/1.5/2.0/2.5 (6)<br>0.78 | 5.1/2.1/5.7/6.4 (10)<br>0.48 | 1.1/0.7/1.1/2.0 (1)<br>0.89 | 2.0/4.2/0.7/0.3 (4)<br>0.70 |
| 14<br>ref: 2bbw (GP5) | 2.6/1.5/2.9/5.9 (2)<br>0.74 | 3.5/2.5/3.9/6.4 (1)<br>0.59 | 2.7/1.3/3.1/5.9 (7)<br>0.79 | 1.6/2.9/0.6/0.2 (2)<br>0.77 |
| 15<br>ref: 2cdn (ADP-1) | 1.9/1.1/2.0/2.6 (1)<br>0.89 | 6.4/3.8/6.9/5.8 (4)<br>0.43 | 1.3/0.9/1.4/2.2 (1)<br>0.86 | 0.5/0.5/0.5/0.2 (1)<br>0.89 |
| 15<br>ref: 2cdn (ADP-2) | 1.1/0.7/1.2/2.8 (1)<br>0.79 | 2.2/1.1/2.6/5.9 (1)<br>0.68 | 0.7/0.7/0.8/2.3 (1)<br>0.90 | 0.4/0.6/0.4/0.2 (1)<br>0.95 |
| 16<br>ref: 5x6l (AP5) | 2.1/2.2/2.0/1.9 (1)<br>0.82 | 4.3/1.9/4.8/5.1 (7)<br>0.59 | 1.3/1.0/1.3/2.0 (1)<br>0.92 | 1.1/2.2/0.5/0.2 (1)<br>0.90 |
| 17<br>ref: 5xz2 (AP5) | 2.1/2.4/2.0/2.2 (4)<br>0.82 | 4.3/2.9/4.6/5.1 (8)<br>0.56 | 1.4/1.7/1.3/2.2 (1)<br>0.92 | 2.3/5.1/0.6/0.2 (2)<br>0.67 |
| 18<br>ref: 5ycf (AP5) | 2.5/1.2/2.7/2.4 (10)<br>0.84 | 5.4/3.4/5.9/5.3 (7)<br>0.58 | 1.3/1.4/1.2/2.1 (1)<br>0.95 | 0.6/0.7/0.5/0.2 (1)<br>0.97 |
| 19<br>ref: 5x6k (AP5) | 2.4/2.2/2.5/2.4 (8)<br>0.64 | 4.9/2.6/5.3/5.3 (6)<br>0.56 | 2.0/1.0/2.1/2.0 (1)<br>0.90 | 0.5/0.6/0.5/0.2 (1)<br>1.00 |
| 20<br>ref: 5ycd (AP5) | 2.4/1.6/2.6/2.5 (35)<br>0.74 | 5.1/3.1/5.5/5.5 (2)<br>0.58 | 1.4/0.8/1.4/2.1 (1)<br>0.92 | 0.5/0.8/0.4/0.2 (1)<br>0.97 |
| 21<br>ref: 5g3y (ADP-1) | 1.8/1.1/1.9/5.4 (1)<br>0.80 | 3.1/2.1/3.3/7.5 (1)<br>0.56 | 0.7/1.0/0.7/1.8 (1)<br>0.88 | 0.4/0.7/0.3/0.2 (1)<br>1.00 |
| 21<br>ref: 5g3y (ADP-2) | 2.2/2.4/2.1/2.6 (2)<br>0.82 | 5.8/2.7/6.3/7.4 (7)<br>0.37 | 0.9/0.7/0.8/1.8 (1)<br>1.00 | 0.5/0.5/0.5/0.2 (1)<br>1.00 |
| 22<br>ref: 5g3z (AP5) | 2.4/3.2/2.1/2.5 (27)<br>0.73 | 5.3/2.4/5.8/6.9 (4)<br>0.50 | 1.2/0.8/1.3/1.9 (1)<br>0.91 | 0.5/0.6/0.4/0.2 (1)<br>0.93 |
| % native-like confs. **top-1** | 9/26 (34.6%) | 1/26 (3.8%) | 24/26 (92.3%) | 20/26 (76.9%) |
| % native-like confs. **top-5** | 17/26 (65.4%) | 1/26 (3.8%) | 24/26 (92.3%) | 25/26 (96.2%) |
| % native-like confs. **top-10** | 21/26 (80.8%) | 2/26 (7.7%) | 24/26 (92.3%) | 25/26 (96.2%) |
| % native-like confs. **Any rank** | 24/26 (92.3%) | 2/26 (7.7%) | 24/26 (92.3%) | 25/26 (96.2%) |

**Table S7. Number of productive conformers per complex for the $HM_{gEDES}$ ensembles.** The second column reports the number of receptor conformers, out of 101, that individually yield a pose with $RMSD_{BM}$ ≤ 2.5 Å. The best $RMSD_{BM}$ reported in the 3rd column is the minimum over all poses of the complex, which differs from the value tabulated in Table S6, that one being the representative of the cluster containing the best native-like solution. The two sites of the dual-ligand systems 2, 9, 15 and 21 are listed separately, since each was docked as a separate complex.

| System (ligand) | # (out of 101) | Best $RMSD_{BM}$ [Å] |
|---|---|---|
| ***1 (AP5)*** | 8 | 2.05 |
| ***2 (ADP)*** | 31 | 1.23 |
| ***2 (AMP)*** | 10 | 1.84 |
| ***3 (AP5)*** | 7 | 1.86 |
| ***4 (AP5)*** | 5 | 2.11 |
| ***5 (AP5)*** | 6 | 1.86 |
| ***6 (AP5)*** | 6 | 2.23 |
| ***7 (AP5)*** | 8 | 1.84 |
| ***8 (AP5)*** | 7 | 1.82 |
| ***9 (ADP)*** | 30 | 1.41 |
| ***9 (AMP)*** | 10 | 1.68 |
| ***10 (AP5)*** | 4 | 2.17 |
| ***11 (AMP)*** | 12 | 1.87 |
| ***12 (G5P)*** | - | 4.53 |
| ***13 (AP5)*** | 7 | 1.91 |
| ***14 (GP5)*** | - | 2.63 |
| ***15 (ADP-1)*** | 6 | 1.90 |
| ***15 (ADP-2)*** | 45 | 1.06 |
| ***16 (AP5)*** | 1 | 2.11 |
| ***17 (AP5)*** | 2 | 2.15 |
| ***18 (AP5)*** | 1 | 2.46 |
| ***19 (AP5)*** | 1 | 2.38 |
| ***20 (AP5)*** | 1 | 2.38 |
| ***21 (ADP-1)*** | 29 | 1.23 |
| ***21 (ADP-2)*** | 10 | 2.14 |
| ***22 (AP5)*** | 4 | 2.16 |
| ***Summary*** | Median: 7 | 2.04 ± 0.64 |

## References


(1) Sievers, F.; Higgins, D. G. The Clustal Omega Multiple Alignment Package. In *Multiple Sequence Alignment*; Katoh, K., Methods in Molecular Biology; Ed. Springer US: New York, NY, **2021**; Vol. 2231, pp 3–16. https://doi.org/10.1007/978-1-0716-1036-7_1

(2) Brown, N. P.; Leroy, C.; Sander, C. MView: A Web-Compatible Database Search or Multiple Alignment Viewer. *Bioinformatics* **1998**, *14* (4), 380–381. https://doi.org/10.1093/bioinformatics/14.4.380

(3) Gouet, P. ESPript/ENDscript: Extracting and Rendering Sequence and 3D Information from Atomic Structures of Proteins. *Nucleic Acids Research* **2003**, *31* (13), 3320–3323. https://doi.org/10.1093/nar/gkg556

(4) The UniProt Consortium; Bateman, A.; Martin, M.-J.; Orchard, S.; Magrane, M.; Adesina, A.; Ahmad, S.; Bowler-Barnett, E. H.; Bye-A-Jee, H.; Carpentier, D.; Denny, P.; Fan, J.; Garmiri, P.; Gonzales, L. J. D. C.; Hussein, A.; Ignatchenko, A.; Insana, G.; Ishtiaq, R.; Joshi, V.; Jyothi, D.; Kandasaamy, S.; Lock, A.; Luciani, A.; Luo, J.; Lussi, Y.; Marin, J. S. M.; Raposo, P.; Rice, D. L.; Santos, R.; Speretta, E.; Stephenson, J.; Totoo, P.; Tyagi, N.; Urakova, N.; Vasudev, P.; Warner, K.; Wijerathne, S.; Yu, C. W.-H.; Zaru, R.; Bridge, A. J.; Aimo, L.; Argoud-Puy, G.; Auchincloss, A. H.; Axelsen, K. B.; Bansal, P.; Baratin, D.; Batista Neto, T. M.; Blatter, M.-C.; Bolleman, J. T.; Boutet, E.; Breuza, L.; Gil, B. C.; Casals-Casas, C.; Echioukh, K. C.; Coudert, E.; Cuche, B.; De Castro, E.; Estreicher, A.; Famiglietti, M. L.; Feuermann, M.; Gasteiger, E.; Gaudet, P.; Gehant, S.; Gerritsen, V.; Gos, A.; Gruaz, N.; Hulo, C.; Hyka-Nouspikel, N.; Jungo, F.; Kerhornou, A.; Mercier, P. L.; Lieberherr, D.; Masson, P.; Morgat, A.; Paesano, S.; Pedruzzi, I.; Pilbout, S.; Pourcel, L.; Poux, S.; Pozzato, M.; Pruess, M.; Redaschi, N.; Rivoire, C.; Sigrist, C. J. A.; Sonesson, K.; Sundaram, S.; Sveshnikova, A.; Wu, C. H.; Arighi, C. N.; Chen, C.; Chen, Y.; Huang, H.; Laiho, K.; Lehvaslaiho, M.; McGarvey, P.; Natale, D. A.; Ross, K.; Vinayaka, C. R.; Wang, Y.; Zhang, J. UniProt: The Universal Protein Knowledgebase in 2025. *Nucleic Acids Research* **2025**, *53* (D1), D609–D617. https://doi.org/10.1093/nar/gkae1010

(5) Webb, B.; Sali, A. Comparative Protein Structure Modeling Using MODELLER. *CP in Bioinformatics* **2016**, *54* (1). https://doi.org/10.1002/cpbi.3

(6) Li, D.; Liu, M. S.; Ji, B. Mapping the Dynamics Landscape of Conformational Transitions in Enzyme: The Adenylate Kinase Case. *Biophysical Journal* **2015**, *109* (3), 647–660. https://doi.org/10.1016/j.bpj.2015.06.059

(7) Giulini, M.; Reys, V.; Teixeira, J. M. C.; Jiménez-García, B.; V. Honorato, R.; Kravchenko, A.; Xu, X.; Versini, R.; Engel, A.; Verhoeven, S.; Bonvin, A. M. J. J. HADDOCK3: A Modular and Versatile Platform for Integrative Modeling of Biomolecular Complexes. *J. Chem. Inf. Model.* **2025**, *65* (13), 7315–7324. https://doi.org/10.1021/acs.jcim.5c00969
